# Structure–Processing–Property Relationships in PVOH/PBAT Blends Plasticized with Dipentaerythritol, ε‑Caprolactone, and Water

**DOI:** 10.1021/acsomega.6c07788

**Published:** 2026-10-12

**Authors:** Sakib Khan, Abu Saifullah, Zhongyi Zhang, Hom N. Dhakal

**Affiliations:** School of Electrical and Mechanical Engineering, 6697 University of Portsmouth, Portsmouth PO1 3DJ, U.K.

## Abstract

Water-soluble poly­(vinyl alcohol) (PVOH) offers high tensile strength and an excellent oxygen barrier, but its dense hydrogen-bond network places its melting point close to its decomposition temperature, making it difficult to melt-process. Poly­(butylene adipate-*co*-terephthalate) (PBAT) is melt-processable and ductile but has low strength and dimensional stability that restrict its use. To combine their strengths, PBAT/PVOH blends (10–40 wt % PBAT) were melt-compounded with a 20 wt % ternary plasticizer of dipentaerythritol, ε-caprolactone, and water in equal parts, benchmarked against a water-only control at 40 wt % PBAT. Across FTIR, XRD, DSC, steady-shear and oscillatory rheology, DMA, tensile, SEM, AFM, TGA, and gas-barrier measurements, the ternary plasticizer governs the structure–property relationship by redistributing the PVOH hydrogen-bond network rather than breaking it; it broadens and shifts the O–H stretching envelope, suppresses PVOH crystallinity and recrystallization, depresses the glass transition by 17–27 °C, and broadens the relaxation spectrum, with no cross-linking or covalent grafting. Composition sets the phase topology—dispersed PBAT domains at 20–30 wt % and a cocontinuous structure at 40 wt %—while the plasticizer controls interfacial adhesion, converting the weakly bonded, void-laden interfaces of the water-only control into well-adhered ones. Among the series, the 20 wt % blend (PBAT-20) gives the best stiffness–toughness balance (modulus ≈ 0.23 GPa, ultimate tensile strength ≈ 20 MPa) together with an oxygen transmission rate of ≈6.4 cm^3^ m^–2^ day^–1^ for a 400 μm film, equivalent to ≈102 cm^3^ m^–2^ day^–1^ when normalized to a 25 μm thickness. The water-vapor transmission rate of the same film remains high (6.06 g m^–2^ day^–1^), so the films reported here are suited to oxygen-sensitive but moisture-tolerant applications rather than to moisture-critical ones. These findings position ternary hydrogen-bond engineering as a reaction-free, melt-processing route to property-tunable PBAT/PVOH films for biodegradable flexible packaging.

## Graphical abstract

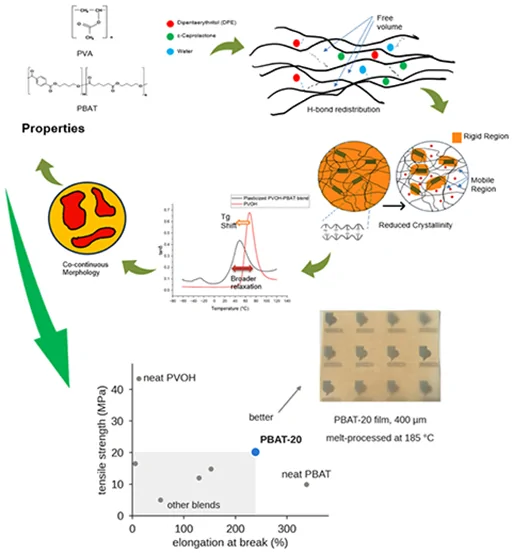

## 1 Introduction

Flexible packaging remains dominated by polyolefins because they are inexpensive, easily processed, and durable, but their persistence after disposal has made biodegradable replacements a priority. Among biodegradable polyesters, poly­(butylene adipate-*co*-terephthalate) (PBAT) is one of the most widely adopted because it combines flexibility, film-forming ability, and compostability. Its low modulus and tensile strength, however, restrict its use in load-bearing or dimensionally stable applications, and foamed PBAT additionally suffers from low melt strength, shrinkage, and weak compressive performance.^1,2^ Raising the stiffness and barrier properties of PBAT without sacrificing its degradability therefore requires a second component that contributes mechanical reinforcement and gas-barrier function.

Poly­(vinyl alcohol) (PVOH) provides exactly aforementioned attributes. Its hydroxyl-rich backbone forms a dense, three-dimensional hydrogen-bonded network that delivers high tensile strength and an oxygen barrier among the best of any synthetic polymer. The same network is also the source of PVOH’s principal processing limitation: its melting temperature lies close to its decomposition range, leaving a narrow thermal-processing window and poor melt processability that historically confined PVOH to aqueous casting.^3,4^ Eliminating the solvent route requires lowering the effective melting point and melt viscosity of PVOH, which is achieved by plasticization prior to or during melt blending.^4,5^

Plasticization of PVOH is best described as a redistribution of hydrogen bonding rather than the destruction of it. The hydroxyl groups of PVOH partition into three segments: intramolecular, intermolecular, and free O–H and relative proportions of the latter can be read directly from the breadth and position of the infrared O–H envelope.^6,7^ Small hydrogen-bonding molecules do not cleave covalent bonds; they compete for donor and acceptor sites, converting PVOH–PVOH contacts into PVOH–plasticizer contacts. The result is increased free volume, greater segmental mobility, a lower glass transition, and a broadened distribution of relaxation times.^6,8,9^ Because the same hydrogen-bond population controls crystallinity, melt and solid-state relaxation, surface energy, and the temperature at which dehydration-driven degradation begins, the way a plasticizer rearranges that network governs essentially every property measured in this study.^9–11^

Most PVOH-based blends rely on water and low-molar-mass polyols mainly glycerol, with sorbitol, xylitol, and poly­(ethylene glycol) as common alternatives as the primary plasticizers, because these molecules hydrogen-bond efficiently with PVOH hydroxyls and raise chain mobility.^6,11,12^ Their efficiency is, however, accompanied by drawbacks for thermal processing: glycerol in particular volatilizes and migrates at extrusion temperatures, which limits its industrial use and produces property drift on storage.^12^ Comparative studies of glycerol and diethanolamine show that the choice of polyol changes the strength–toughness balance and the homogeneity of the resulting film, indicating that plasticizer architecture, not merely loading, sets the property outcome.^13^ These observations motivate plasticizer systems that combine multiple hydrogen-bond functionalities with reduced volatility.^14^

Combining PBAT with plasticized PVOH is attractive because each polymer offsets a weakness of the other: PBAT supplies ductility and melt processability, while PVOH supplies stiffness, strength, and barrier. Earlier work established the morphology and mechanics of melt-processed PBAT/PVOH parts and the dependence of phase evolution on composition.^15^ Increasing PVOH content in PBAT/PVOH blends raises tensile strength that is for neat PBAT near 14.5 MPa rising to roughly 53 MPa for an 80 wt % PVOH blend, while accelerating degradation in water and soil.^1^ In foamed systems, thermal processing combined with supercritical-CO_2_ foaming yields high specific compressive strength and reduced shrinkage.^2^ The central obstacle in every case is compatibility: PVOH is strongly polar and PBAT comparatively hydrophobic, so phase separation is difficult to avoid unless interfacial hydrogen bonding is deliberately managed. Hydrogen-bond regulation and molecular complexation were recently used to produce interface-compatible PVOH/PBAT blown films by melt processing, with a raised PVOH glass transition, improved PBAT dispersion, a water contact angle of 100.1°, tensile strength of 44.0 MPa, and soil disintegration within 25 days which is the evidence that PBAT/PVOH blends need not be limited to coarse two-phase morphologies.^16^

End-of-life behavior depends on the disposal pathway and should not be overstated. PBAT and PVOH degrade most rapidly in wastewater (25.6 and 32.2% in 30 days, respectively) and far more slowly in seawater, so a single “biodegradable” label is inadequate for materials that move through treatment plants, rivers, and coastal waters.^16^ Circularity offers a complementary metric: thermoplastic-starch/PVOH blends retain mechanical performance over ten extrusion cycles, in contrast to the rapid loss of properties on reprocessing of PLA and PHB, which places PVOH-containing blends within a recyclability framework as well as a composability one.^17^

To the best of the authors’ knowledge, no PBAT/PVOH system plasticized with the combination of dipentaerythritol (DPE), ε-caprolactone (ε-CL), and water has previously been reported. Historically, plasticization of PVOH has centered on water–polyol mixtures and rarely exceeded binary formulations. Conventional water–polyol plasticization is single-target: water, glycerol, sorbitol, and poly­(ethylene glycol) all compete for the same PVOH–PVOH hydroxyl contacts, so they raise bulk PVOH mobility while leaving the PBAT–PVOH interface, which is the property-limiting feature of this blend, unaddressed; the most effective of them also volatilize or migrate at extrusion temperature. The strategy adopted here is instead role-divided, each component being selected for a distinct hydrogen-bonding function. DPE is a six-hydroxyl, low-volatility polyol that redistributes the bulk PVOH network and persists through compounding at 185 °C. ε-CL is ester-bearing and is expected to partition preferentially to the PBAT polyester phase and to the interface, an interfacial-coupling function that no polyol provides. Water acts as a transient, mobile hydrogen-bond modulator that raises chain mobility during processing. Because composition and total plasticizer loading are held constant in the matched pair PBAT-40 and PBAT-40-W, the contribution of the nonvolatile DPE and ε-CL is isolated experimentally rather than inferred. This work is based on the hypothesis that the DPE/ε-CL/water ternary plasticizer modifies the hydrogen-bonding network of PVOH in PBAT/PVOH blends more effectively than water alone. This redistribution of hydrogen bonding is expected to control the resulting crystallinity, melt and solid-state relaxation, phase morphology, mechanical performance, surface energy, and thermal degradation behavior.

## 2 Experimental Section

### 2.1 Materials

Poly­(butylene adipate-*co*-terephthalate) (PBAT; Ecoflex F) was obtained from BASF. Subpartially hydrolyzed PVOH (degree of hydrolysis ≈ 72.5 mol %, weight-average molar mass ≈ 13 kg mol^–1^; grade BC-05) was supplied by Chang Chun Petrochemical (CCP). Dipentaerythritol (DPE, 80+%) and ε-caprolactone (ε-CL, 97% purity) were purchased from Thermo Scientific and Sigma-Aldrich respectively and used as received. PBAT pellets and PVOH powder were dried under vacuum at 60 °C for 24 h before compounding to remove moisture and limit hydrolytic degradation. The 72.5 mol % hydrolysis grade contains approximately 27.5 mol % residual vinyl acetate units, which interrupt the regular hydroxyl-rich sequence of PVOH, reduce intermolecular hydrogen bonding and chain packing, and therefore lower crystallinity and melting temperature relative to fully hydrolyzed grades, which is consistent with the low PVOH melting endotherm in Section 3.3.^6,18^

### 2.2 Melt Blending and Film Preparation

Blends were prepared by melt compounding. The process is physical plasticization, not reactive blending: no initiator or coupling agent was added, and FTIR showed no new covalent bands (Section 3.1). PVOH, PBAT, and the plasticizer were compounded in a HAAKE Rheomix PolyLab QC internal mixer with roller rotors at 185 °C and 50 rpm for 10 min. The total plasticizer content was fixed at 20 wt % for every blend: in the ternary series it was DPE, ε-CL, and water in equal parts (≈6.7 wt % of the formulation each), and in the water-only control (PBAT-40-W) it was water alone. The total plasticizer content was fixed at 20 wt % to depress the melting point of PVOH below its decomposition onset and enable melt processing, a value consistent with the 15–30 wt % range conventionally reported for melt compounding of PVOH.^19–21^ The PBAT fraction was varied from 10 to 40 wt % with PVOH making up the balance (Table 1). ε-CL was charged as a 95 wt % solution in toluene; toluene was removed during compounding, pressing, and drying. ε-CL acted as a monomeric internal plasticizer, and ring-opening polymerization was neither intended nor detected by FTIR. The melt was transferred to a preheated steel frame, compression-molded at 185 °C and 10 MPa for 3 min and cooled between air-cooled platens to films ≈400 μm thick, from which specimens were die-cut.^5,12^

**Table 1:** Composition of the PBAT/PVOH Formulations^a^

Sample	PBAT (wt %)	PVOH (wt %)	Plasticizer (wt %)	Plasticizer composition
Neat PVOH	0	100	0	none
PBAT-10	10	70	20	DPE + ε-CL + water (equal parts)
PBAT-20	20	60	20	DPE + ε-CL + water (equal parts)
PBAT-30	30	50	20	DPE + ε-CL + water (equal parts)
PBAT-40	40	40	20	DPE + ε-CL + water (equal parts)
PBAT-40-W	40	40	20	water only (control)
Neat PBAT	100	0	0	none

a Blend codes denote the PBAT weight fraction; PVOH makes up the balance and the plasticizer is fixed at 20 wt% (ternary series: dipentaerythritol, ε-caprolactone, and water in equal parts; control PBAT-40-W: water only). DPE = dipentaerythritol; ε-CL = ε-caprolactone.

### 2.3 Conditioning

Before testing, specimens were dried at 85 °C for 1 h to standardize moisture content and give reproducible properties. This removes free and loosely bound water; water hydrogen-bonded to PVOH and to the plasticizer is retained, consistent with the low-temperature endotherm observed by DSC (Section 3.3) and with the role of nonfreezing, bound water in PVOH plasticization.^8^

### 2.4 Characterization

#### 2.4.1 Fourier-Transform Infrared Spectroscopy (FTIR)

Attenuated-total-reflectance spectra were recorded on a Thermo Nicolet Nexus 870 spectrometer with a ZnSe ATR accessory over 4000–500 cm^–1^ (2 cm^–1^ resolution, 64 scans); backgrounds were subtracted automatically.

#### 2.4.2 X-ray Diffraction (XRD)

Patterns were collected on a Malvern Panalytical Aeris diffractometer (Cu Kα, λ = 0.154 nm; 40 kV, 15 mA) over 2θ = 5–50° at 2° min^–1^ with a 0.02° step. Crystallinity and crystallite sizes were obtained from the patterns.

#### 2.4.3 Differential Scanning Calorimetry (DSC)

Transitions were measured on a TA Instruments DSC 25 (≈6 mg, N_2_ at 50 mL min^–1^) by heating and cooling between −50 and 200 °C at 10 °C min^–1^; Tg, Tm, and Tc were taken from the second heating and cooling (3 replications for each sample). Crystallinity of each semicrystalline phase was calculated from the second-heating melting enthalpy normalized to the corresponding phase weight fraction and to the melting enthalpy of the fully crystalline polymer (eq 3, Section 3.3), for which reference values of ΔHm° = 114 J g^–1^ for PBAT and ΔHm° = 138.6 J g^–1^ for PVOH were used.

#### 2.4.4 Thermogravimetric Analysis (TGA)

Stability was measured on a TA Instruments TGA 550 (≈8 mg, 30–500 °C at 10 °C min^–1^, N_2_ at 60 mL min^–1^; n = 3 per material). Derivative (DTG) curves were computed from the mass signal and are plotted on the same temperature axis as the mass-loss curves. Characteristic temperatures are reported in Table 7.

#### 2.4.5 Rheology

Melt rheology was measured on a TA Instruments Discovery HR-3 with a 25 mm parallel-plate geometry (1 mm gap) at 185 °C. Steady-shear flow sweeps (0.01–1000 s^–1^) gave the viscosity–shear-rate curves fitted with the Carreau–Yasuda model over the artifact-free range (Section 3.5); small-amplitude oscillatory frequency sweeps (0.1–100 rad s^–1^) within the linear viscoelastic region (1% strain) gave G′, G″, and |η*| (Section 3.6).

#### 2.4.6 Dynamic Mechanical Analysis (DMA)

A TA Instruments Q800 in dual-cantilever mode tested 30 × 5 mm bars from −60 to 120 °C at 3 °C min^–1^ under N_2_ (1 Hz, 15 μm amplitude); E′, E″, and tan δ were recorded.

#### 2.4.7 Tensile Testing

Properties were measured per ASTM D638–14 on a Zwick/Roell Z010 (10 kN load cell) with Type V dog-bone at 23 °C and 50% RH and 10 mm min^–1^. Five specimens per formulation were tested; mean ultimate tensile strength, Young’s modulus, and elongation at break (±SD) are reported.

#### 2.4.8 Water Contact Angle

Static angles were measured on a KSV CAM-101 goniometer by depositing a 2 μL distilled-water droplet and recording the angle immediately at five locations per film (23 ± 2 °C); means ± SD are reported.

#### 2.4.9 Scanning Electron Microscopy (SEM)

Fracture surfaces were imaged on a Zeiss Evo 10 microscope at 15 kV after gold sputter-coating. Tensile-fractured and cryo-fractured (liquid-nitrogen) surfaces were examined to assess the dispersion of PBAT in the PVOH matrix and the effect of plasticization on morphology.

#### 2.4.10 Atomic Force Microscopy (AFM)

Topography and phase contrast were examined on a Park Systems NX10 in tapping mode in air (silicon cantilever, ≈40 N m^–1^, ≈300 kHz), recording height and phase images simultaneously to distinguish PBAT- and PVOH-rich domains. Three areas were scanned per film at 40–50 μm scan size. The areal roughness parameters Sa, Sq and Sz were evaluated over the entire scanned area following ISO 25178 and are reported as means ± SD over the three areas; the height images in Figure 7b carry labeled x, y and z axes, and a representative mean-centered line profile extracted from each scan is plotted on a common vertical scale in Figure 7c.

#### 2.4.11 Gas-Barrier Properties

Oxygen transmission rate (OTR) was measured on a MOCON OX-TRAN 2/40 per ASTM D3985 at 23 °C and 50% RH (50 cm^2^ area; number of samples = 3). Water-vapor transmission rate (WVTR) was measured by the gravimetric cup method (ASTM E96) with silica-gel desiccant at 50% RH and 23 °C (number of samples = 3).

## 3 Results and Discussion

### 3.1 FTIR: Hydrogen-Bond Redistribution

Infrared spectroscopy resolves the three hydroxyl populations of PVOH and is used here to test whether the ternary plasticizer redistributes, rather than removes, hydrogen bonding. Neat PVOH shows the broad O–H stretching envelope at 3200–3550 cm^–1^ (hydrogen-bonded O–H), C–H stretching near 2900 cm^–1^, and the C–O stretch near 1090 cm^–1^; the crystallinity-sensitive band at 1141 cm^–1^ tracks the ordered fraction of the matrix.^7^ Neat PBAT contributes its ester carbonyl near 1715 cm^–1^ and aromatic C=C near 1505 cm^–1^.^22^

Across the blends, the carbonyl and O–H bands of both polymers are retained, no new covalent bands appear, and no characteristic absorptions are lost, confirming physical blending rather than chemical reaction. Increasing PBAT content from PBAT-20 to PBAT-30 raises the relative intensity of the 1715 cm^–1^ ester carbonyl and lowers that of the PVOH O–H band as illustrated in Figure 1a, consistent with the changing phase ratio. The diagnostic change attributable to plasticization is in the O–H envelope: relative to neat PVOH it broadens and its maximum shifts, the signature of PVOH–PVOH contacts being partly replaced by PVOH–plasticizer (DPE and water) and interfacial PVOH–PBAT interactions. A red shift indicates a population of stronger, more cooperative multidonor hydrogen bonds introduced by the multihydroxyl DPE, whereas a blue-shifted shoulder indicates weaker, less cooperative O–H···O contacts where rigid PBAT/ε-CL segments interrupt the self-associated PVOH network; both contributions broaden the envelope simultaneously. The accompanying reduction in the 1141 cm^–1^ crystallinity band corroborates loss of ordered PVOH packing on plasticization.^7,11^

**Figure 1:**
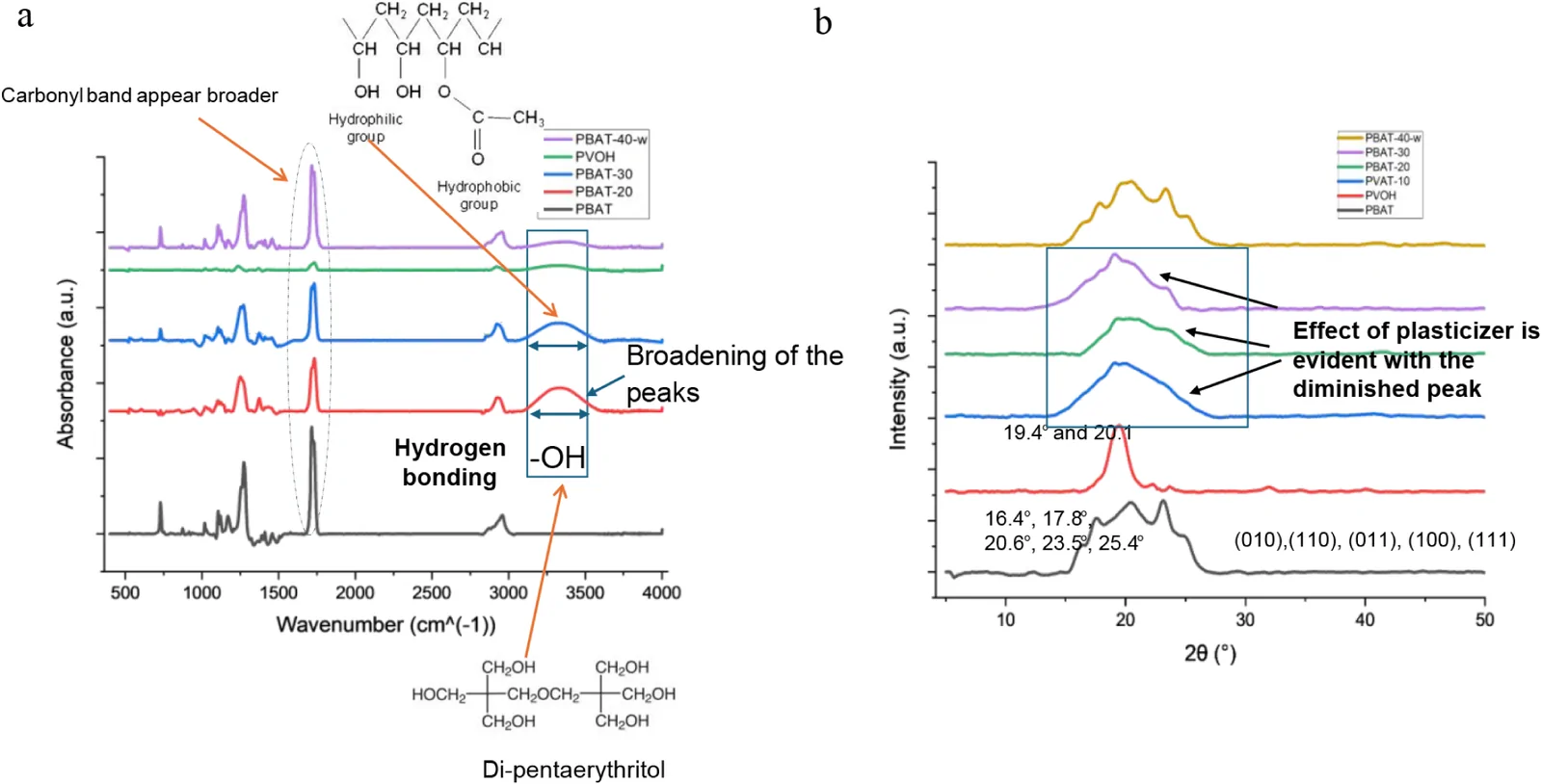
(a) ATR-FTIR absorbance spectra (4000–500 cm^–1^; ordinate in absorbance units, a.u., curves vertically offset for clarity) of neat PBAT, neat PVOH, the ternary-plasticized blends (PBAT-10 to PBAT-40), and the water-only control (PBAT-40-W). Diagnostic bands: hydrogen-bonded O–H (3200–3550 cm^–1^), C–H (≈2900 cm^–1^), ester C=O (≈1715 cm^–1^), the PVOH crystallinity-sensitive band (1141 cm^–1^), and C–O (≈1090 cm^–1^). (b) XRD patterns (2θ = 5–50°, Cu Kα) of the same materials, showing the PVOH reflection near 2θ ≈ 19–20° and PBAT reflections at 2θ ≈ 17–25°. Derived crystallinities and crystallite sizes are given in Table 2.

Two controls help separate plasticization from covalent cross-linking. In chemically cross-linked PVOH systems, glutaraldehyde or succinyl chloride consumes free hydroxyl groups and introduces acetal or ester linkages, usually accompanied by new characteristic FTIR bands and increased stiffness. The absence of these additional bands in the present blends indicates no spectroscopic evidence of covalent network formation; therefore, the observed O–H band broadening and shifts are attributed mainly to physical redistribution of hydrogen bonding.^7^ The water-only blend, PBAT-40-W, also shows a broadened and shifted O–H envelope, but the effect is weaker than in the full ternary plasticized system. This confirms that water acts as an internal plasticizer, while DPE and ε-CL further enhance hydrogen-bond disruption and chain mobility.^8,9^ These assignments are consistent with previous reports showing that polyol-type plasticizers redistribute PVOH hydrogen bonding and reduce crystallinity.

### 3.2 XRD: Suppressed and Constrained Crystallinity

Neat PVOH shows the broad semicrystalline reflection at 2θ ≈ 19–20° from hydrogen-bond-stabilized chain packing; neat PBAT shows reflections at 2θ ≈ 17.3, 17.9, 20.5, 23.1, and 24.9° assigned to its aliphatic–aromatic crystal.^22,23^ The blends contain reflections from both phases. In PVOH-rich PBAT-10 the PVOH reflection remains dominant but is broader and weaker than in neat PVOH as shown in Figure 1b, indicating reduced packing order; as PBAT increases to PBAT-40 the PBAT reflections strengthen yet remain broader than in neat PBAT, indicating crystallization constrained within the blend.^15,24^

To place these qualitative trends on a quantitative footing, the degree of crystallinity (*X*_*c*_) and the apparent crystallite size (*D*) were extracted from each pattern. After confirming a flat background, the patterns were deconvoluted over 2θ = 10–32° into a set of Gaussian crystalline reflections, fixed near the characteristic PVOH and PBAT positions, superimposed on a single broad amorphous halo; the crystallinity was then obtained from eq 1:

1 $$X_{c}\;(\%)=\frac{I_{c}}{I_{c}+I_{a}}\times 100$$

where *I*_c_ is the summed area of the crystalline reflections and *I*_a_ is the area of the amorphous halo (the same fitting protocol was applied to every sample, all fits giving *R* ^*2*^ ≥ 0.996).^25–27^ Apparent crystallite sizes were calculated from the fitted peak widths using the Scherrer equation, eq 2:

2 $$D=\frac{K\lambda }{\beta \cos\theta }$$

with shape factor *K* = 0.9, Cu Kα wavelength λ = 0.15406 nm, and β the fitted full width at half-maximum (in radians) of the principal PVOH (101) reflection at ∼19.5° and the well-isolated PBAT (100) reflection at ∼23.1°;the instrumental contribution was negligible relative to the very broad polymer reflections. The resulting values are collected in Table 2.

**Table 2:** Degree of Crystallinity (*X_c_*) from Peak-Deconvolution Integration and Apparent Crystallite Size (*D*, Scherrer Equation) for the Principal PVOH (101) and PBAT (100) Reflections

Sample	*X*_c_ (%)	*D*, PVOH (101) (nm)	*D*, PBAT (100) (nm)
**Neat PVOH**	77	5.0	—
PBAT-10	16	3.8	2.7
PBAT-20	31	5.0	3.6
PBAT-30	24	6.6	5.7
PBAT-40	25	—^a^	4.9
PBAT-40-W	31	6.3	7.7
**Neat PBAT**	36	—	6.2

a PVOH (101) overlaps the PBAT ∼ 20.5° reflection in PBAT-rich blends and was not reliably separated. The PBAT (100) reflection at ∼23.1° is the most isolated and is the more reliable size metric (uncertainty ≈ ±1 nm).

The quantitative results mirror the visual trends. Neat PVOH is by far the most ordered material (*X*_c_ ≈ 77%), and neat PBAT is moderately crystalline (*X*_c_ ≈ 36%); every blend falls well below both parent polymers. Crystallinity collapses to ≈16% in the PVOH-rich PBAT-10 and remains in the ≈24–31% range from PBAT-20 to PBAT-40 as displayed in Table 2, confirming that blending and plasticization disrupt the dense hydrogen-bonded PVOH lattice without allowing the PBAT phase to crystallize freely. The apparent crystallite sizes are correspondingly small for both phases (∼3–8 nm) and follow the same pattern: the broadest reflections, and hence the smallest coherent domains (PBAT (100) ≈ 2.7–3.6 nm), occur in the fully plasticized PVOH-rich blends, whereas the larger PBAT domains develop as PBAT content rises and are largest in the neat polymer and the water-only control. The ∼5 nm PVOH domains are consistent with values reported for semicrystalline PVOH films.^11^ Together, the reduced *X*_c_ and the small *D* values give a direct, quantitative measure of the suppressed and constrained crystallization inferred from the peak shapes.

The plasticizer is the controlling variable. The fully plasticized blends show broader, weaker reflections and correspondingly lower crystallinity and smaller crystallites than the water-only blend, while PBAT-40-W shows sharper PVOH reflections (*X*_c_ ≈ 31%; *D*_PBAT(100)_ ≈ 7.7 nm versus ≈ 4.9 nm for plasticized PBAT-40) consistent with more efficient, direct PVOH–PVOH association.^11^ The suppression of sharp PVOH peaks in the ternary blends parallels the behavior of low-hydrolysis PVOH grades, in which residual acetate units and disrupted stereoregularity broaden the crystalline reflections; here the DPE/ε-CL/water system reproduces this effect physically by diluting the hydrogen-bond density available for crystallization. Eleven The XRD trends agree with the DSC results (Section 3.3) and with the reduced 1141 cm^–1^ FTIR band, all of which indicate that ternary plasticization lowers crystalline perfection and drives constrained crystallization.

### 3.3 Second-Heating DSC: Glass Transition and Suppressed PVOH Recrystallization

Second-heating thermograms remove prior thermal history and report the reorganized phases as illustrated in Figure 2a. Neat PBAT shows a glass transition below −30 °C and a melting peak at 122 °C with ΔHm = 17.56 J g^–1^. The crystallinity of the PBAT phase was calculated using^28^

3 $$X_{c,\mathrm{PBAT}}\;(\%)=\frac{\Delta H_{m}-\Delta H_{cc}}{w_{\mathrm{PBAT}}\Delta H_{m}^{0}}\times 100$$

**Figure 2:**
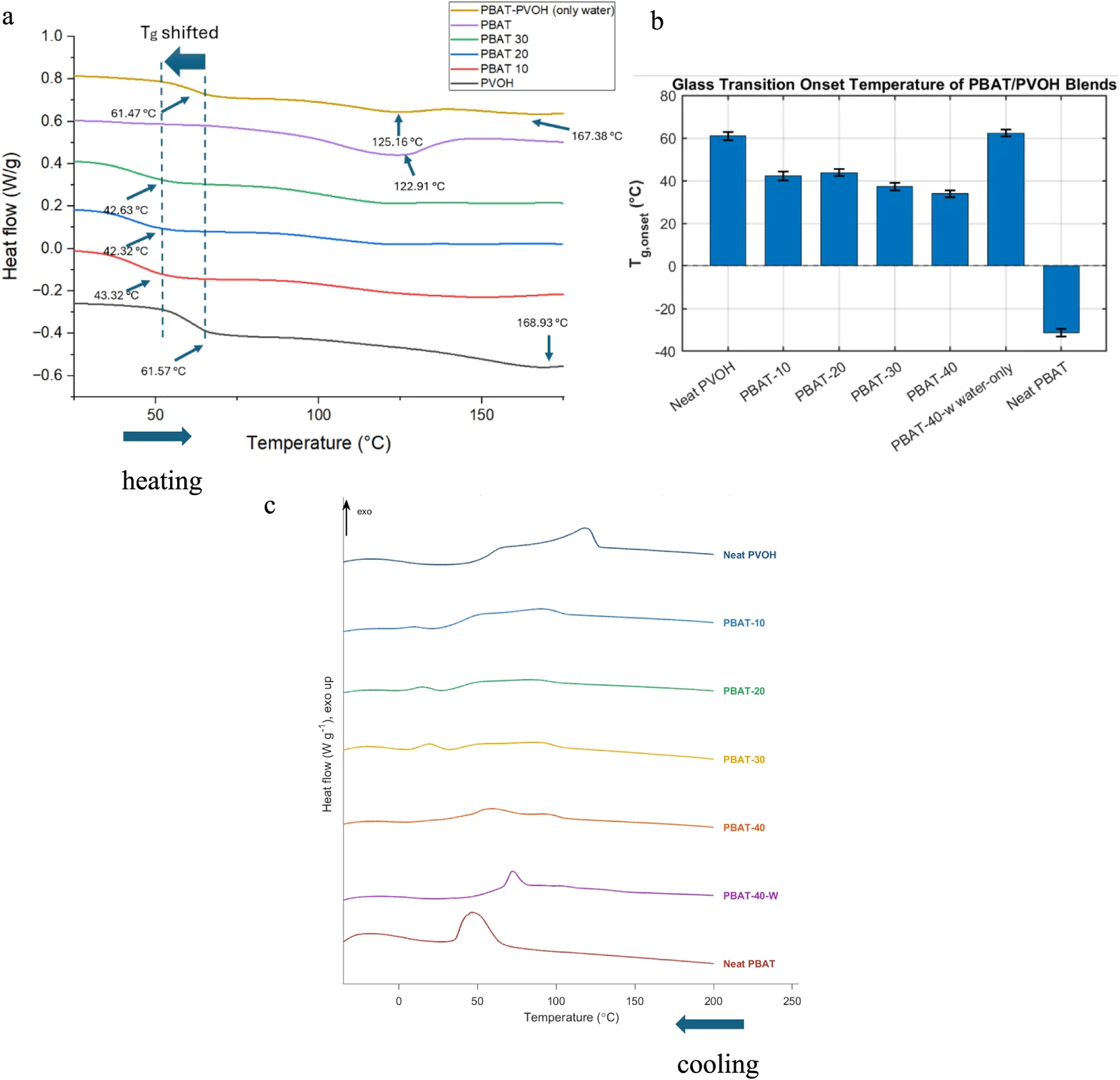
(a) Second-heating DSC thermograms (10 °C min^–1^, N_2_; endothermic response downward, as indicated by the arrow) of the neat polymers and blends; glass transition, melting peak, and melting enthalpy are marked. Values of Tg, Tm and crystallinity are collected in Table 3. (b) Mean glass-transition onset for every material, from three replicate scans (means ± SD). (c) DSC melt-crystallization (cooling) thermograms of the neat polymers and blends, recorded from 200 to −40 °C at 10 °C min^–1^ (exo up). Neat PVOH crystallizes at Tc ≈ 118 °C and neat PBAT at Tc ≈ 47 °C; the principal exotherm of the blends shifts from ≈90 °C (PBAT-10) to ≈59 °C (PBAT-40), and a weak low-temperature exotherm near 15 °C in the PVOH-rich blends marks fractionated crystallization of the dispersed PBAT phase. The ternary PBAT-40 crystallizes ≈14 °C below the water-only control PBAT-40-W (Tc ≈ 73 °C).

Where, *X_c_* is the degree of crystallinity of the PBAT phase (%), Δ*H_m_* is the melting enthalpy obtained from the DSC thermogram (J g), Δ*H_cc_* is the cold-crystallization enthalpy (J g) when present, *w*_PBAT_ is the weight fraction of PBAT in the blend, and $\Delta H_{m}^{0}$ is the melting enthalpy of 100% crystalline PBAT.^28^ A value of Neat PVOH shows a glass-transition onset near 61 °C and a melting peak at 166.9 °C with ≈9% crystallinity as displayed in Table 3.

**Table 3:** Glass-Transition Onset (Tg, Onset), Melting Peak Temperature (Tm), and Degree of Crystallinity (Xc) of the Neat Polymers and Blends, Determined from the Second-Heating DSC Scan (10 °C min^–1^, N_2_)­^a^

Sample	Tg,onset (°C)	PBAT Tm (°C)	Xc PBAT (%)	PVOH Tm (°C)	Xc PVOH (%)
**Neat PVOH**	60.93 ± 1.23	—	—	166.86 ± 5.45	8.98 ± 0.74
PBAT-10	42.19 ± 0.84	121.26 ± 2.67	12.81 ± 0.89	—	—
PBAT-20	43.80 ± 0.91	119.48 ± 3.20	12.63 ± 0.37	—	—
PBAT-30	37.10 ± 0.87	117.38 ± 4.54	12.37 ± 0.67	—	—
PBAT-40	33.95 ± 0.67	118.20 ± 1.81	9.04 ± 1.32	—	—
PBAT-40-W	62.25 ± 1.10	122.11 ± 4.89	6.53 ± 1.14	166.64 ± 2.33	4.00 ± 1.50
**Neat PBAT**	–31.33±0.45	121.89 ± 1.12	15.40 ± 2.16	—	—

a Values are means ± SD.

In the ternary blends the PVOH glass-transition onset falls well below 61 °C—42.2, 43.8, 37.1, and 33.9 °C for PBAT-10 to PBAT-40 as illustrated in Figure 2b and a downward shift that quantifies the increased mobility of the PVOH-rich phase produced by the plasticizer. The PBAT melting peak persists in all blends but moves to 117–121 °C with reduced normalized enthalpy; PBAT crystallinity falls from ≈12.8% (PBAT-10) to ≈9.0% (PBAT-40), so although total melting enthalpy rises with PBAT fraction, the efficiency of PBAT crystallization declines as the local environment is increasingly plasticized.

The absence of the PVOH melting endotherm provides strong evidence that the plasticizer disrupted the crystalline ordering of the PVOH phase. The 166.9 °C endotherm of neat PVOH is not resolved in any ternary blend, indicating that PVOH does not recrystallize appreciably once its hydrogen-bond network is redistributed. The water-only control isolates this effect: in PBAT-40-W the glass-transition onset returns to 62.3 °C and a small PVOH endotherm reappears at 166.6 °C (≈4.0% PVOH crystallinity), while PBAT melts at 122.1 °C with ≈6.5% crystallinity. Water alone therefore permits partial PVOH recrystallization, whereas DPE and ε-CL suppress it which direct the thermal confirmation that the ternary system, not water, governs the PVOH crystalline phase. This dependence of melting and recrystallization on plasticizer type is consistent with reported polyol effects on PVOH thermal transitions.^10^

In the PVOH-rich blends (PBAT-10 to PBAT-30), where PBAT forms the dispersed minor phase, a weak exotherm appears near 15 °C as shown in Figure 2c well below the bulk crystallization of neat PBAT (≈47 °C). This high-undercooling event is the signature of fractionated crystallization of the finely dispersed PBAT domains: subdivided into many droplets that outnumber the available heterogeneous nuclei, most domains are nucleus-free and crystallize only at much larger undercooling, so the dispersed-phase exotherm splits off from the bulk value and shifts to low temperature.^10^ Because the phenomenon is sensitive to the number and size of the dispersed domains, it also serves as a qualitative DSC fingerprint of the state of dispersion.^10^ Consistent with outcome, the low-temperature exotherm is confined to the PVOH-rich compositions and is replaced, once PBAT becomes cocontinuous at 40 wt % (Section 3.8), by the broad main exotherm at Tc ≈ 59 °C with only a high-temperature shoulder near 90 °C.

The absolute crystallinities returned by DSC and XRD differ substantially: neat PVOH gives ≈9% by DSC but ≈77% by XRD, and neat PBAT ≈ 15% versus ≈36%, yet the two are complementary rather than contradictory, because they measure different observables on samples in different thermal states. XRD reports a structural quantity, the fraction of material ordered enough to produce coherent Bragg reflections in the as-molded film, whereas DSC reports a thermodynamic quantity, the melting enthalpy of the second-heating scan normalized to that of a perfect crystal (eq 3); there is no general numerical conversion between the two.^27^ Three factors account for the size of the gap here. First, the measurements probe different states: XRD samples the as-cast film, while the DSC crystallinity is taken after the thermal history has been erased, so for a polymer such as PVOH that recrystallizes poorly once its hydrogen-bond network is redistributed the reformed crystal fraction and hence the DSC value is intrinsically lower than the order retained in the original film.^10^ Second, the small, defective crystallites indicated by the Scherrer sizes (≈3–8 nm, Table 2) still diffract but melt with less enthalpy per unit mass than ideal crystals, biasing the DSC value below the XRD value.^10^ Third, the DSC scale depends on the reference enthalpy ΔHm° and the XRD scale on the crystalline–amorphous peak deconvolution, so each absolute value carries method-specific uncertainty.^10,27^ Importantly, the two techniques agree on every trend that matters: both show that blending and plasticization sharply suppress PVOH order relative to the neat polymer, and both isolate the water-only control as retaining more crystalline character than the ternary blends, a residual PVOH endotherm and a higher XRD crystallinity in PBAT-40-W. We therefore use XRD for absolute crystalline content and crystallite size and DSC for the transitions and the suppression trend, comparing the two based on relative change across the series rather than absolute magnitude.

### 3.4 Steady-Shear Rheology and Carreau–Yasuda Modeling

Steady-shear flow curves were recorded at 185 °C from 0.01 to 10^3^ s^–1^ (Figure 3a) and fitted with the Carreau–Yasuda model up to 160 s^–1^. Each curve shows a low-shear Newtonian plateau followed by shear thinning. From the fits, the zero-shear viscosity η_0_ places neat PVOH (≈3.9 × 10^4^ Pa·s) well above every blend and neat PBAT lowest (≈1.0 × 10^3^ Pa·s), with the ternary blends in between (8.6 × 10^3^ to 1.3 × 10^4^ Pa·s) and the water-only control lower still (5.6 × 10^3^ Pa·s; Table 4). η_0_ does not vary monotonically with PBAT content: PBAT-20 is the lowest of the blends (8.6 × 10^3^ Pa·s) while PBAT-40 is among the highest (1.3 × 10^4^ Pa·s), so composition alone does not set the low-shear viscosity. The plasticizer does: replacing the ternary additive with water (PBAT-40-W, η_0_ ≈ 5.6 × 10^3^ Pa·s) appears to lower η_0_ below that of the fully plasticized PBAT-40. This ordering should not be read as evidence that water is the more effective plasticizer, because it rests on the two poorest Carreau–Yasuda fits in the set (R^2^ = 0.94 and 0.76) and is reversed by the oscillatory measurement; the point is taken up below and resolved in Section 3.5.

**Figure 3:**
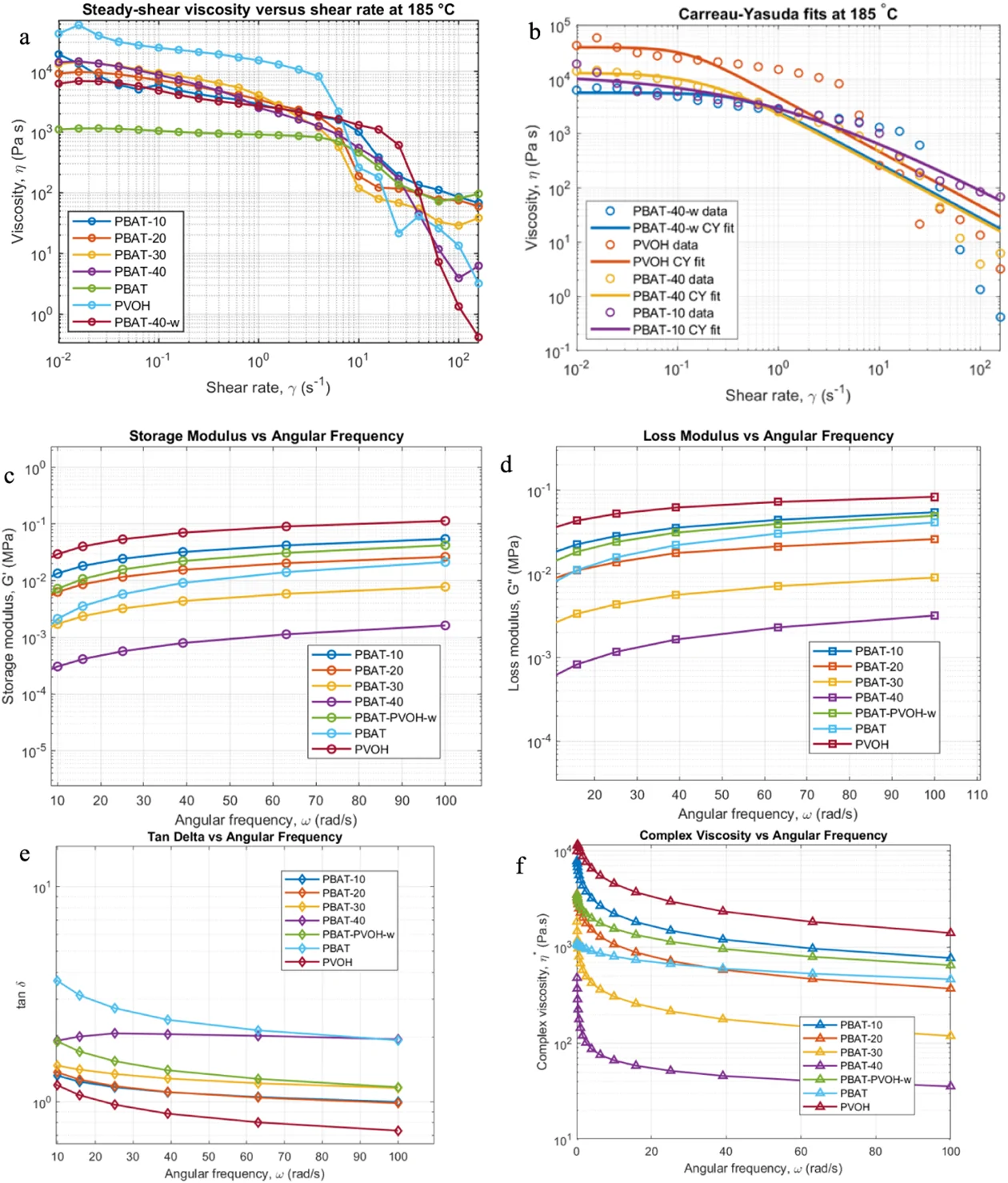
Melt rheology at 185 °C. (a) Steady-shear viscosity versus shear rate for all samples; (b) Carreau–Yasuda fits to γ̇ ≤ 160 s^–1^ for selected samples (PVOH, PBAT-10, PBAT-40, PBAT-40-W), with fitted parameters listed in Table 4. (c–f) Small-amplitude oscillatory frequency sweeps within the linear viscoelastic region (1% strain), shown as (c) storage modulus G′, (d) loss modulus G″, (e) loss tangent tan δ, and (f) complex viscosity |η*| versus angular frequency for the neat polymers and blends.

**Table 4:** Carreau–Yasuda Parameters Obtained from the Steady-Shear Flow Curves at 185 °C, Fitted over the Artifact-Free Range γ̇ ≤ 160 s^–1^: Zero-Shear Viscosity η_0_, Characteristic Relaxation Time λ, and Coefficient of Determination R^2^ of the Fit on Logarithmic Axes^a^

Sample	η_0_ (Pa·s)	λ (s)	R^2^ (log)
Neat PVOH	3.9 × 10^4^	8.37	0.874
PBAT-10	1.26 × 10^4^	1.19	0.965
PBAT-20	8.63 × 10^3^	2.34	0.968
PBAT-30	1.23 × 10^4^	4.56	0.971
PBAT-40	1.32 × 10^4^	5.30	0.936
PBAT-40-W	5.64 × 10^3^	2.03	0.760
Neat PBAT	1.02 × 10^3^	0.30	0.974

a Only η_0_ and λ are interpreted here; the fits for neat PVOH and PBAT-40-W are the poorest in the set (R^2^ = 0.87 and 0.76), so their η_0_ values are the least reliable and the oscillatory data of Section 3.5 are used in preference where the two disagree.

The model is shown in the eq 4.

4 $$\eta \left(\mathrm{\gamma ̇}\right)=\eta_{\infty }+\left(\eta_{0}-\eta_{\infty }\right){[1+{\left(\lambda \mathrm{\gamma ̇}\right)}^{a}]}^{n-1/a}$$

with characteristic time λ, power-law index *η*, and transition breadth a.^29,30^ Of the fitted parameters, η_0_ and λ are set by the low-to-moderate-shear data and are the interpretable descriptors here. The reciprocal of λ marks the onset of shear thinning: neat PBAT has the shortest relaxation time (λ ≈ 0.30 s) and PVOH the longest (λ ≈ 8.4 s), so the PVOH melt begins thinning at the lowest shear rate (Figure 3b), as expected for an entangled, hydrogen-bonded network. Across the blends λ rises with PBAT content (1.2, 2.3, 4.6, and 5.3 s for PBAT-10 to PBAT-40), consistent with an additional long-time relaxation contributed by the deformable PBAT-rich domains of an immiscible melt, which lengthens as the dispersed phase grows toward cocontinuity.^14^

The power-law index and transition breadth require caution. The fits return n ≈ 0 for every sample, with a scattered between 0.5 and 4.3; these are not material constants but a consequence of the high-shear data. In the present geometry the reliable parallel-plate window appears to end near ≈10–60 s^–1^, beyond which slip and edge effects become likely.^10,29^ The symptom is unambiguous: above this range the apparent viscosity falls below 1 Pa·s for several samples (3.2 Pa·s for PVOH and 0.4 Pa·s for PBAT-40-W within the fitted range), far below any plausible melt viscosity at 185 °C, and the curves turn nonmonotonic at the highest rates. The apparent log–log slope steepens past −1 (≈−1.7 for PVOH, ≈−2.8 for PBAT-40-W), whereas the Carreau–Yasuda terminal slope cannot exceed −1 for *n* ≥ 0; the optimizer is therefore driven to its lower bound in n, and the fit quality degrades precisely for the samples with the steepest spurious decay (R^2^ = 0.87 for PVOH and 0.76 for PBAT-40-W, versus 0.94–0.97 for the others). Restricting the fit to the monotonic, physically plausible window restores conventional behavior: a Carreau fit of PBAT-40 to ≈40 s^–1^ returns η_0_ ≈ 1.5 × 10^4^ Pa·s with n ≈ 0.47, essentially reproducing the full-range η_0_ and confirming that the n ≈ 0 result is an artifact of the corrupted high-shear data rather than a property of the melt. Because the low-shear η_0_ is recovered independently of the high-shear branch, we interpret only η_0_ and λ and, where a cross-check is useful, refer to the oscillatory complex viscosity (Section 3.5), which is less sensitive to these flow instabilities.^31^

Two features confirm that the blends are not simple viscosity averages. First, the PBAT-10 to PBAT-40 flow curves cross as shear rate increases rather than ranking monotonically with PBAT content, so the additive package and phase arrangement reshape the relaxation spectrum rather than only the mean viscosity which consistent with reports that matching the plasticized-PVOH and PBAT melt viscosities enables uniform blending through interfacial coupling.^16^ Second, the steady-shear fit places PBAT-40 (η_0_ ≈ 1.3 × 10^4^ Pa·s) above PBAT-40-W (η_0_ ≈ 5.6 × 10^3^ Pa·s), whereas the oscillatory complex viscosity reverses this ordering at low frequency (Section 3.5); because the lower-quality fits for these two samples (R^2^ = 0.94 and 0.76) make their steady-shear η_0_ the less reliable estimate, we treat the oscillatory data as the more dependable measure of the low-deformation melt response. Either way, the ternary additive and the water-only control differ in how the blend stores and releases structure during flow through phase continuity and the balance of PBAT-rich and PVOH-rich relaxation rather than only in mean viscosity.^15^

For processing, the reliable low-shear parameters suffice: PVOH and the PVOH-rich blends carry the highest η_0_ and longest λ, so they need the most plastication torque but begin thinning at the lowest shear rates, while the ternary PBAT-40, which has the lowest complex viscosity at low frequency (Section 3.5), flows most readily. We base this on the oscillatory data rather than on the steady-shear η_0_ of PBAT-40-W, whose fit is the least reliable in the set. The high-shear viscosities should not be used to set injection or die shear rates until the measurements are repeated with edge-fracture and slip controls using either a smaller gap, cone-partitioned plate, or capillary rheometry.^10,30^

### 3.5 Oscillatory Rheology: Broadening of the Relaxation Spectrum

Frequency sweeps over 0.1–100 rad s^–1^ probe the relaxation-time distribution of the melt (Figure 3c–f). At the lowest accessible frequency (ω = 0.1 rad s^–1^) neat PVOH has the highest complex viscosity of any sample, |η*| ≈ 1.0 × 10^4^ Pa·s, with a loss tangent tan δ ≈ 6.7; neat PBAT is far less viscous, |η*| ≈ 1.1 × 10^3^ Pa·s, but markedly more dissipative, tan δ ≈ 16.6. PVOH is therefore the more viscous, more strongly associated phase, while molten PBAT is the softer, predominantly viscous polyester. Because interfacial modifiers and plasticizers act most strongly on low-frequency relaxation in immiscible blends, this regime is the most sensitive representation of plasticization.

Incorporating PBAT and the plasticizer depresses the low-frequency complex viscosity relative to neat PVOH, and the magnitude of the drop scales with the degree of plasticization. At 0.1 rad s^–1^, |η*| falls from ≈ 1.0 × 10^4^ Pa·s (PVOH) to ≈ 6.4 × 10^3^ Pa·s (PBAT-10), ≈ 3.5 × 10^3^ Pa·s (PBAT-20), and ≈ 1.8 × 10^3^ Pa·s (PBAT-30), and reaches its lowest value in the fully plasticized PBAT-40, |η*| ≈ 4.8 × 10^2^ Pa·s that is more than an order of magnitude below neat PVOH, because the ternary additive most effectively increases free volume and chain mobility; comparable mobility gains are reported for ≈30 wt % water in PVOH, which raises free volume and lowers *T*_g_.^8^ The water-only control behaves differently: PBAT-40-W retains a much higher low-frequency viscosity, |η*| ≈ 3.5 × 10^3^ Pa·s, comparable to PBAT-20 and far above the fully plasticized PBAT-40, indicating that water alone is the weaker plasticizer of the melt and that the nonvolatile DPE and ε-CL are responsible for the largest viscosity reduction. The loss tangent distinguishes the two further. At 0.1 rad s^–1^ the ternary PBAT-30 and PBAT-40 show the lowest tan δ of all samples (≈0.9 and ≈0.7), i.e., a predominantly elastic, network-like response, whereas PBAT-40-W shows tan δ ≈ 3.8 and PBAT-10 and neat PVOH retain tan δ ≈ 6, more viscous responses dominated by uncoupled chain relaxation. The combination of the lowest |η*| with the lowest tan δ in the ternary high-PBAT blends is the melt-state signature of a compatibilized, cocontinuous morphology, in which the excess low-frequency elasticity arises from the interfacial area and from coupling between the phases rather than from a high bulk viscosity; this interpretation is consistent with the established use of low-frequency oscillatory elasticity to detect interfacial coupling and compatibilization in immiscible blends.^15,16^

At high frequency (ω = 100 rad s^–1^) the imposed deformation is too fast for interfacial or large-scale rearrangement, so the response reflects local segmental dynamics: the |η*| curves draw together over a far narrower range (≈4 × 10^1^ to 1.4 × 10^3^ Pa·s) and the loss tangents converge toward order unity (tan δ ≈ 0.7–2), reflecting the shift from a relaxation-controlled to a segmental, more elastic response shared by all compositions. The strong frequency dependence at low ω, followed by convergence at high ω, indicates a broad relaxation-time distribution in the blend. This behavior is consistent with partial phase coupling among PBAT-rich, PVOH-rich and plasticizer-rich regions, and agrees with the broadened DMA α-relaxation discussed in Section 3.6.

### 3.6 Dynamic Mechanical Analysis: α-Relaxation Depression and Broadening

DMA resolves the solid-state relaxations and quantifies plasticization through the position, height, and breadth of the PVOH α-transition (Figure 4). In the glassy state at −60 °C all samples are stiff and the storage modulus does not order monotonically with composition: PBAT-10 is the stiffest of all (E′ ≈ 0.71 GPa), followed by neat PBAT (≈0.49 GPa), neat PVOH (≈0.45 GPa), PBAT-20 (≈0.44 GPa) and PBAT-30 (≈0.37 GPa), with the two 40 wt % PBAT materials lowest (PBAT-40-W ≈ 0.22 GPa and PBAT-40 ≈ 0.21 GPa) as shown in Figure 4a. The plasticization effect emerges above the PBAT glass transition: by 25 °C the modulus has dropped to ≈0.47 GPa (PBAT-10), ≈0.39 GPa (PVOH), ≈0.26 GPa (PBAT-20), ≈0.17 GPa (PBAT-30) and ≈0.11 GPa (PBAT-40), while the water-only control PBAT-40-W falls furthest, to ≈0.055 GPa. Above the PVOH α-relaxation every sample reaches a low, rubbery plateau. The room-temperature ranking broadly tracks the tensile moduli of Section 3.7, and the steep room-temperature drop in the high-PBAT and water-only materials shows the PVOH network progressively losing load-bearing integrity as PBAT and plasticizer increase.

**Figure 4:**
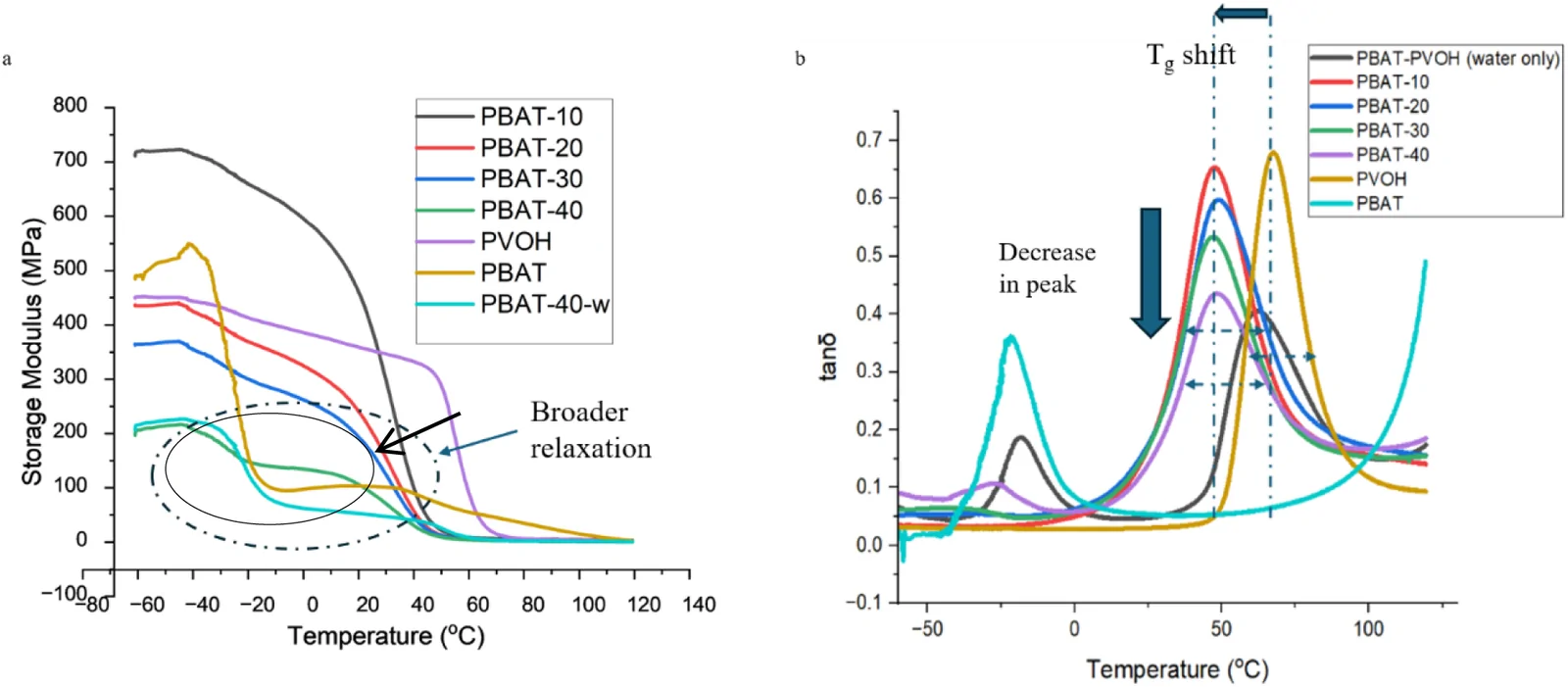
Dynamic mechanical response of the neat polymers and blends in tension (1 Hz, 3 °C min^–1^, −60 to 120 °C): (a) storage modulus E′ and (b) loss tangent tan δ versus temperature. The PBAT glass transition (≈−20 °C) and the PVOH α-relaxation are marked on each trace.

The tan δ spectra contains two relaxations as illustrated in Figure 4b. The low-temperature peak at ≈−22 °C (tan δ ≈ 0.36 in neat PBAT) is the PBAT glass transition; it remains near the same temperature on blending because PBAT forms the dispersed or cocontinuous PBAT-rich phase, although it is largely masked in the blends by the dominant PVOH α-transition. The high-temperature peak is the PVOH α-transition, sharpest and highest in neat PVOH at ≈66 °C (tan δ ≈ 0.68). In the water-only control PBAT-40-W it falls to ≈57 °C (tan δ ≈ 0.39), and in the ternary blends it shifts lower still and broadens, with the peak centered near ≈46–48 °C across PBAT-10 to PBAT-40. Within the ternary plasticized series, the peak height progressively decreases with increasing PBAT content, falling from approximately 0.65 for PBAT-10 and 0.60 for PBAT-20 to 0.53 for PBAT-30 and 0.44 for PBAT-40 reflecting the gradual reduction of the load-bearing PVOH-rich fraction. The downward shift of the PVOH α-relaxation from ≈66 °C (neat) to ≈57 °C (water-only) to ≈46 °C (ternary PBAT-40) is a direct measure of progressive plasticization, and the further ≈9–11 °C drop between PBAT-40-W and the ternary blends isolates the additional mobility contributed by DPE and ε-CL beyond water alone.

The DMA α-relaxation of neat PVOH (≈66 °C, taken at the tan δ maximum) lies just above the calorimetric onset of 61 °C (Section 3.3) because the two techniques probe the transition on different time scales that is why the dynamic measurement at finite frequency reports a slightly higher apparent *T*_g_ than the calorimetric onset, so the two values are complementary rather than contradictory, both placing the neat-PVOH transition in the 60–66 °C range for this partially hydrolyzed grade. Peak broadening, the high-temperature shoulder above the main α-peak in the plasticized samples, and the reduced peak height at higher PBAT fraction together indicate a distribution of segmental mobilities and dynamic coupling between PVOH-rich and PBAT-rich domains through plasticizer-rich interfacial regions. The narrower, higher-temperature tan δ peak of PBAT-40-W shows that water alone leaves more of the PVOH–PVOH network intact, whereas the lower, broader peaks of the ternary blends mark a more fully redistributed network with increased free volume. These results align with FTIR (redistributed hydrogen bonding) and XRD/DSC (reduced crystallinity) and with reports linking PVOH hydration to its dynamic-mechanical response.^31^

### 3.7 Tensile Behavior and Fracture Morphology

The tensile response is governed by the volume fractions of the stiff PVOH-rich and soft PBAT-rich phases and by the quality of the interface between them (Figure 5ii–iv; means ± SD, n = 5). The two homopolymers bracket the behavior but, for modulus and strength, not every blend falls between them. Neat PBAT, whose random aliphatic–aromatic architecture suppresses crystallinity, is soft and weak (E = 0.070 ± 0.006 GPa, UTS = 9.9 ± 0.4 MPa) but extremely ductile, drawing to 338 ± 81% elongation. Neat PVOH is the opposite, being stiff and strong (E = 0.339 ± 0.046 GPa, UTS = 43.4 ± 5.0 MPa) but brittle, failing at only 12.7 ± 2.7% strain as its dense hydrogen-bonded network carries load with little plastic flow.

**Figure 5:**
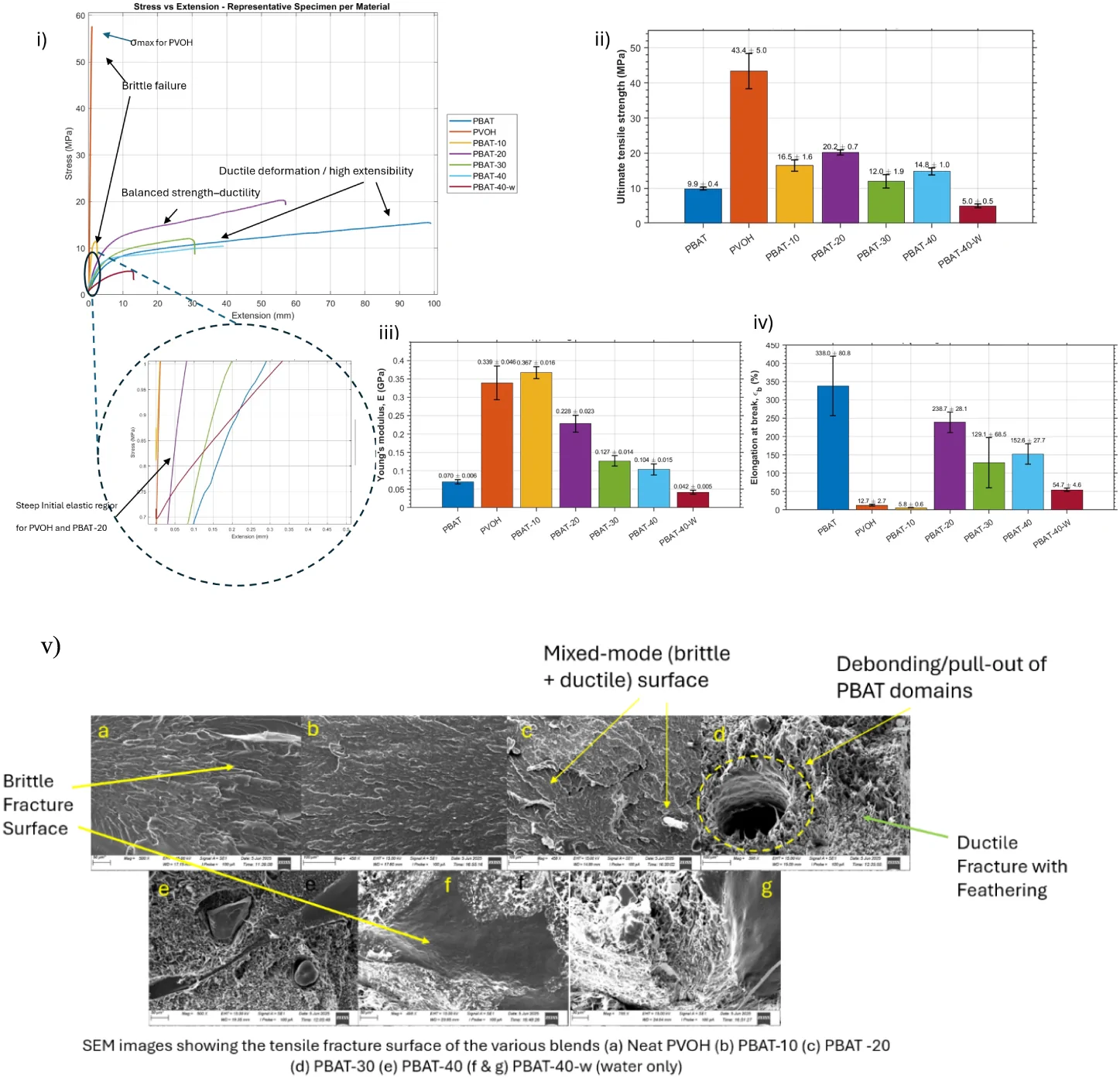
Tensile behavior and fracture morphology of the neat polymers, the ternary-plasticized blends and the water-only control PBAT-40-W (ASTM D638–14, Type V, 10 mm min^–1^, 23 °C, 50% RH). (i) Representative stress–extension curves. (ii) Ultimate tensile strength, (iii) Young’s modulus and (iv) elongation at break, all as means ± SD for *n* = 5; the corresponding values and coefficients of variation are listed in Table 5. (v) Representative tensile-fracture surfaces imaged by SEM at 15 kV: (a) neat PVOH, (b) PBAT-10, (c) PBAT-20, (d) PBAT-30, (e) PBAT-40, and (f, g) two regions of PBAT-40-W showing brittle and ductile (feathered) fracture, respectively. Scale bars and magnifications are marked on each panel.

Within the ternary-plasticized series the modulus falls monotonically with PBAT content, from 0.367 ± 0.016 GPa (PBAT-10) through 0.228 ± 0.023 GPa (PBAT-20) and 0.127 ± 0.014 GPa (PBAT-30) to 0.104 ± 0.015 GPa (PBAT-40), as the soft PBAT-rich phase progressively replaces stiff PVOH-rich material. PBAT-10 is the stiffest blend, comparable to neat PVOH within experimental scatter, and consistent with its highest storage modulus in DMA (Section 3.6), because its 70 wt % PVOH matrix dominates the elastic response; however, the sparse 10 wt % PBAT phase provides no effective energy-dissipating mechanism, so PBAT-10 fails in a brittle manner at very low strain (UTS = 16.5 ± 1.6 MPa, ε_b = 5.8 ± 0.64%). Strength and ductility do not follow the modulus: both peak at PBAT-20, which is the strongest blend (UTS = 20.2 ± 0.7 MPa) and by far the most extensible (ε_b = 238.7 ± 28.1%, second only to neat PBAT). Above 20 wt % PBAT the strength passes through a shallow minimum at PBAT-30 (UTS = 12.0 ± 1.9 MPa; ε_b = 129.1 ± 68.5%, with considerable scatter) and recovers at PBAT-40 (UTS = 14.8 ± 1.0 MPa; ε_b = 152.6 ± 27.7%) as fracture turns more ductile. PBAT-20 therefore offers the best balance of the series, combining the highest blend strength with high ductility and a still-substantial modulus (0.228 GPa), and this combination is responsible for its superior energy absorption relative to the brittle PVOH-rich compositions. The origin of the optimum warrants explicit statement, because it is not predicted by a rule of mixtures: on going from PBAT-10 to PBAT-20 the PVOH content falls from 70 to 60 wt % and the modulus falls by 38%, yet the strength rises by 22% and the elongation at break by a factor of 41. The strength of PBAT-10 is therefore fracture-limited rather than matrix-limited; its PBAT domains are too sparse to blunt or deflect a growing crack, so the specimen fails from a defect before the PVOH-rich matrix approaches its intrinsic strength, consistent with the smooth, featureless fracture surface in Figure 5(v, b). At 20 wt % the dispersed domains reach a density at which crack blunting and matrix shear yielding operate, allowing the same hydrogen-bonded network to sustain a substantially higher stress before failure. Above 20 wt % this gain is progressively offset by dilution of the load-bearing PVOH phase and by the approach to phase inversion at 40 wt % (Section 3.8). PBAT-20 thus lies at the crossover between the onset of effective energy dissipation and the loss of matrix continuity, which is why the optimum appears as a specific composition rather than as a monotonic trend.

The tensile data exhibit property-dependent variability that warrants consideration. Expressed as a coefficient of variation, the modulus is the most reproducible measure across the series at 4–14%, and the ultimate tensile strength lies between 3 and 16%; both are typical of compression-molded films of this thickness. The elongation at break is more widely dispersed (8–53%), and the scatter is concentrated in a single composition. PBAT-30 (±53%) lies closest to the phase-inversion window of ≈30–50 vol % (Section 3.8), where small specimen-to-specimen differences in local topology translate into large differences in the strain at which the PBAT-rich phase begins to carry load, so both brittle and ductile fracture paths are accessible within a single formulation. The remaining compositions, including the PBAT-20 optimum (±12%), scatter no more than neat PBAT itself (±24%). The full data set is given in Table 5.

**Table 5:** Tensile Properties of the Neat Polymers and Blends (ASTM D638-14, Type V, 10 mm min^–1^, 23 °C, 50% RH, N = 5): Young’s Modulus E, Ultimate Tensile Strength UTS, and Elongation at Break εb, Each as Mean ± SD with the Corresponding Coefficient of Variation

Sample	E (GPa)	CV (%)	UTS (MPa)	CV (%)	εb (%)	CV (%)
Neat PBAT	0.070 ± 0.006	**8.6**	9.9 ± 0.4	**4.0**	338.0 ± 80.8	**23.9**
Neat PVOH	0.339 ± 0.046	**13.6**	43.4 ± 5.0	**11.5**	12.7 ± 2.7	**21.3**
PBAT-10	0.367 ± 0.016	**4.4**	16.5 ± 1.6	**9.7**	5.8 ± 0.64	**11.0**
PBAT-20	0.228 ± 0.023	**10.1**	20.2 ± 0.7	**3.5**	238.7 ± 28.1	**11.8**
PBAT-30	0.127 ± 0.014	**11.0**	12.0 ± 1.9	**15.8**	129.1 ± 68.5	**53.1**
PBAT-40	0.104 ± 0.015	**14.4**	14.8 ± 1.0	**6.8**	152.6 ± 27.7	**18.2**
PBAT-40-W	0.042 ± 0.005	**11.9**	5.0 ± 0.5	**10.0**	54.7 ± 4.6	**8.4**

The clearest measure of the plasticizer’s role is the matched pair at 40 wt % PBAT, where composition is held constant and only the plasticizer changes. Removing DPE and ε-CL (PBAT-40-W) degrades every tensile property relative to the ternary PBAT-40: the modulus falls from 0.104 to 0.042 ± 0.005 GPa, the strength from 14.8 to 5.0 MPa, and the elongation from 152.6 to 54.7 ± 4.6%. The water-only control is thus softer, weaker, and less ductile than its ternary counterpart, and weaker and more compliant than neat PBAT itself. This collapse is consistent with the weakly bonded, void-laden PVOH–PBAT interfaces imaged by SEM (Section 3.8): water preserves PVOH–PVOH association (Sections 3.1 and 3.3) but does not bridge the two phases, so the poorly wetted interface debonds under load and caps both stiffness and strength. The nonvolatile, multifunctional DPE and the ester-bearing ε-CL, by contrast, couple the phases and transfer stress across the interface, raising modulus, strength, and ductility simultaneously. That the strength–ductility balance is set by plasticizer architecture and not by loading alone is consistent with reports on plasticized PVOH systems.^9,13^

The tensile-fracture surfaces (Figure 5v, panels a–g) corroborate these trends. Neat PVOH (a) and PBAT-10 (b), the two most brittle compositions, show smooth, near-featureless fracture surfaces: the hydrogen-bonded matrix bears load but dissipates little energy before failure. PBAT-20 (c) shows a markedly rougher, stepped/terraced surface with extensive tearing, the morphology of a tough, ductile fracture consistent with its high elongation. PBAT-30 (d) shows a torn surface with pronounced feathering as the deformable PBAT phase and the redistributed PVOH network dissipate stress, and PBAT-40 (e) shows an embedded PBAT domain on a mixed smooth-and-torn surface, consistent with its cocontinuous topology (Section 3.8). The water-only control fractures in mixed mode: one region of PBAT-40-W (f) is smooth and brittle while another (g) shows ductile feathering, the coexistence reflecting the weak, poorly wetted interfaces of that blend (Section 3.8), where brittle interfacial separation and ductile matrix tearing occur side by side. Overall, PBAT-20 provides the best combination of stiffness, strength, and toughness among the compositions studied.

### 3.8 Cryo-Fracture SEM: Composition- and Plasticizer-Dependent Phase Morphology

Cryo-fracture surfaces of the water-only control and the ternary-plasticized blends are grouped in Figure 6 with matching schematics. Two variables act on the morphology and should be separated. Composition sets the phase topology: at 20–30 wt % PBAT the minor phase forms discrete domains dispersed in a continuous PVOH matrix, whereas at 40 wt % PBAT, which lies within the phase-inversion window of ≈30–50 vol %, the morphology becomes cocontinuous, with interpenetrating PVOH and PBAT networks.^15^ The plasticizer sets the interfacial adhesion at a given topology: the water-only control fractures along weakly bonded interfaces, while the ternary system gives well-adhered ones. PVOH is the continuous matrix at 10–30 wt % PBAT (PVOH = 70, 60, and 50 wt %, respectively) and falls to 40 wt % at PBAT-40, where it equals the PBAT fraction, a 50:50 polymer ratio that places the blend at phase inversion and accounts for its cocontinuous structure (Table 1).

**Figure 6:**
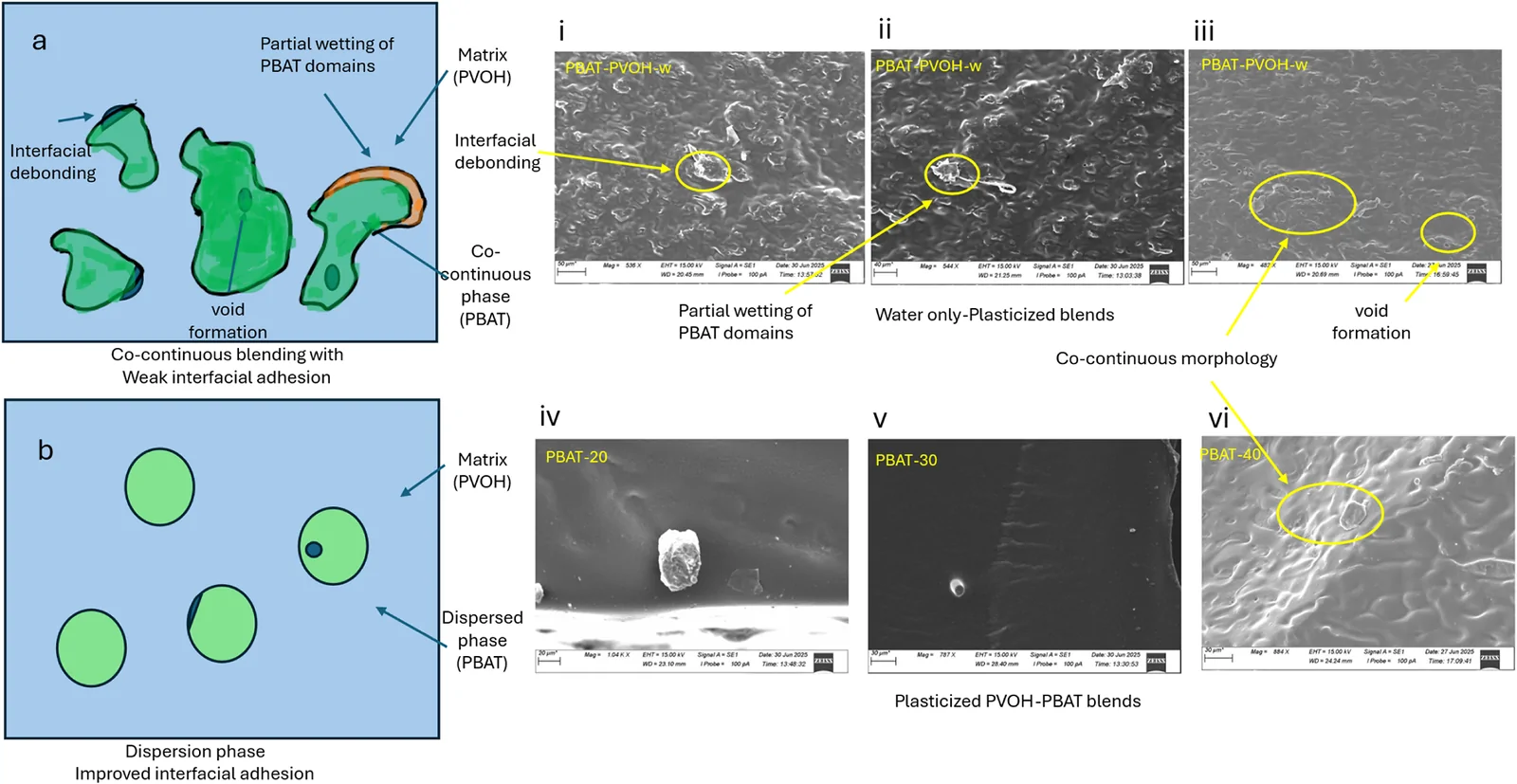
Cryo-fracture SEM micrographs with matching schematics. Panels (i–iii) show the water-only control (PBAT-40-W; 40 wt % PBAT), a cocontinuous morphology with weak interfacial adhesion, evident as partial wetting of the PBAT domains, interfacial debonding and void formation; the accompanying schematic (a) illustrates cocontinuous phases with a poorly bonded interface. Panels (iv–vi) show the ternary-plasticized blends PBAT-20 and PBAT-30, in which PBAT forms dispersed domains in a PVOH matrix, and PBAT-40, which is cocontinuous; all have well-adhered interfaces, as illustrated in schematic (b). Scale bars and magnifications are marked on each panel.

The water-only control (PBAT-40-W; top row, 482–544×, 40–50 μm scale bars) shows large, contiguous PBAT domains together with three features diagnostic of poor interfacial coupling: partial wetting of the PBAT domains, where the matrix incompletely covers the domain surface; interfacial debonding, seen as continuous gaps tracing the domain boundaries; and void formation where domains pulled away during fracture. This is the morphology expected near phase inversion when adhesion is insufficient, so the fracture path follows the weak PVOH–PBAT interface rather than crossing it.^15^ Water alone preserves PVOH–PVOH association (Sections 3.1 and 3.3) and does not bridge the two phases, leaving the interface weak.

In the ternary-plasticized series (bottom row) the topology follows composition. PBAT-20 (1.04k×, 20 μm) and PBAT-30 (787×, 30 μm) show discrete, near-equiaxed PBAT domains seated in a continuous PVOH matrix where the matrix–droplet morphology expected for a minor phase below ≈30–40 vol %. PBAT-40 (884×, 30 μm) is cocontinuous, presenting a rougher, interpenetrating fracture texture rather than isolated domains. Across all three, the interfaces are well bonded: the surfaces lack the clean debonding gaps and pull-out voids of the water-only control, and PBAT-40 fractures with the drawn, ductile texture noted in Section 3.7 rather than by interfacial separation.

This comparison helps separate the effect of composition from that of the plasticizer system. Both the water-only control and the ternary PBAT-40 blend contain 40 wt % PBAT and show a cocontinuous morphology, indicating that the overall phase topology is mainly determined by composition. The key difference lies at the interface. In the water-only blend, the cocontinuous structure is associated with poor interfacial adhesion, partial wetting, debonding and void formation. In contrast, the ternary PBAT-40 blend retains a cocontinuous morphology but shows better adhesion and more ductile interfacial deformation. The DPE/ε-caprolactone/water system therefore acts on interfacial coupling: the multihydroxyl DPE and the ester-bearing ε-CL add hydrogen-bonding and polar contacts that bridge the PVOH and PBAT phases (FTIR, Section 3.1) and lower interfacial tension, strengthening adhesion at every composition and refining the dispersed domains at 20–30 wt % PBAT, without removing the cocontinuity that composition imposes near phase inversion.^15,16^ This is consistent with the rheological signature of interfacial coupling (Sections 3.5 and 3.6) and the stiffness–toughness trade-off in Section 3.7.

### 3.9 Surface Wettability and Hydroxyl Availability

Static water contact angle measures the availability of surface hydroxyls and therefore the surface signature of plasticization (values are means of left/right angles on three films, ±SD). Neat PVOH is strongly hydrophilic (47.3 ± 4.4°) owing to its dense surface hydroxyls, whereas neat PBAT is more hydrophobic (84.3 ± 1.2°). The PBAT value exceeds some literature reports (≈70°), which we attribute to flow-induced surface topography in the melt-processed, compression-molded films that accentuates the intrinsic hydrophobicity through roughness^32,33^ (Section 3.10).

Contact angle increases monotonically with PBAT fraction: 54.1 ± 1.6° (PBAT-10), 72.1 ± 3.1° (PBAT-20), 77.0 ± 2.9° (PBAT-30), and 82.1 ± 3.2° (PBAT-40), tracking the progressive replacement of surface hydroxyls by the more hydrophobic PBAT phase as its loading rises. PBAT-40-W reaches 83.1 ± 2.4°, close to the ternary PBAT-40 and far above neat PVOH, showing that water-only plasticization likewise reduces the availability of surface hydroxyls rather than preserving PVOH’s hydrophilicity.^34^

### 3.10 AFM Surface Topography

AFM three-dimensional height images (Figure 7b; 40–50 μm scans) and the corresponding roughness parameters (Table 6) describe the air-facing surfaces of the films. Neat PVOH is the roughest surface (Sa = 152.6 ± 90.7 nm, Sq = 170.6 ± 98.1 nm), built from large rounded granular mounds separated by deep valleys, the texture of its dense, highly hydrogen-bonded and partly crystalline network; the scatter is as large as the mean is high, so this topography varies markedly from one region of the film to another. Adding PBAT and the ternary plasticizer smooths the surface monotonically: Sa falls from 152.6 nm for neat PVOH through 96.8 ± 22.7 nm (PBAT-10), 78.7 ± 40.2 nm (PBAT-20) and 55.9 ± 5.9 nm (PBAT-30) to 16.3 ± 2.0 nm at PBAT-40, a 9-fold reduction across the series, with Sq following the same order. The maximum height Sz does not track Sa: PBAT-10 records the largest Sz of the whole series (462.0 ± 53.7 nm) despite a midrange Sa, identifying its surface as a smooth matrix carrying a few very tall isolated features rather than a uniformly rough one, as summarized in Table 6.^35,36^

**Table 6:** Areal Surface Roughness of the Neat Polymers and Blends Measured by AFM in Tapping Mode (Three Areas per Film, 40–50 μm Scan Size, 23 °C), Evaluated over the Whole Scanned Area Following ISO 25178: Arithmetic Mean Height Sa, Root-Mean-Square Height Sq, and Maximum Height Sz, Each Given as Mean ± SD over the Three Areas^a^

Sample	Sa (nm)	Sq (nm)	Sz (nm)
Neat PVOH	152.6 ± 90.7	170.6 ± 98.1	433.2 ± 141.4
PBAT-10	96.8 ± 22.7	113.7 ± 24.5	462.0 ± 53.7
PBAT-20	78.7 ± 40.2	85.3 ± 40.5	196.2 ± 37.8
PBAT-30	55.9 ± 5.9	69.1 ± 6.7	291.1 ± 29.5
PBAT-40	16.3 ± 2.0	21.4 ± 3.1	117.3 ± 3.7
PBAT-40-W	116.3 ± 97.0	129.0 ± 100.2	315.0 ± 129.2

a The corresponding height images are shown in Figure 7b and the extracted line profiles in Figure 7c.

**Figure 7:**
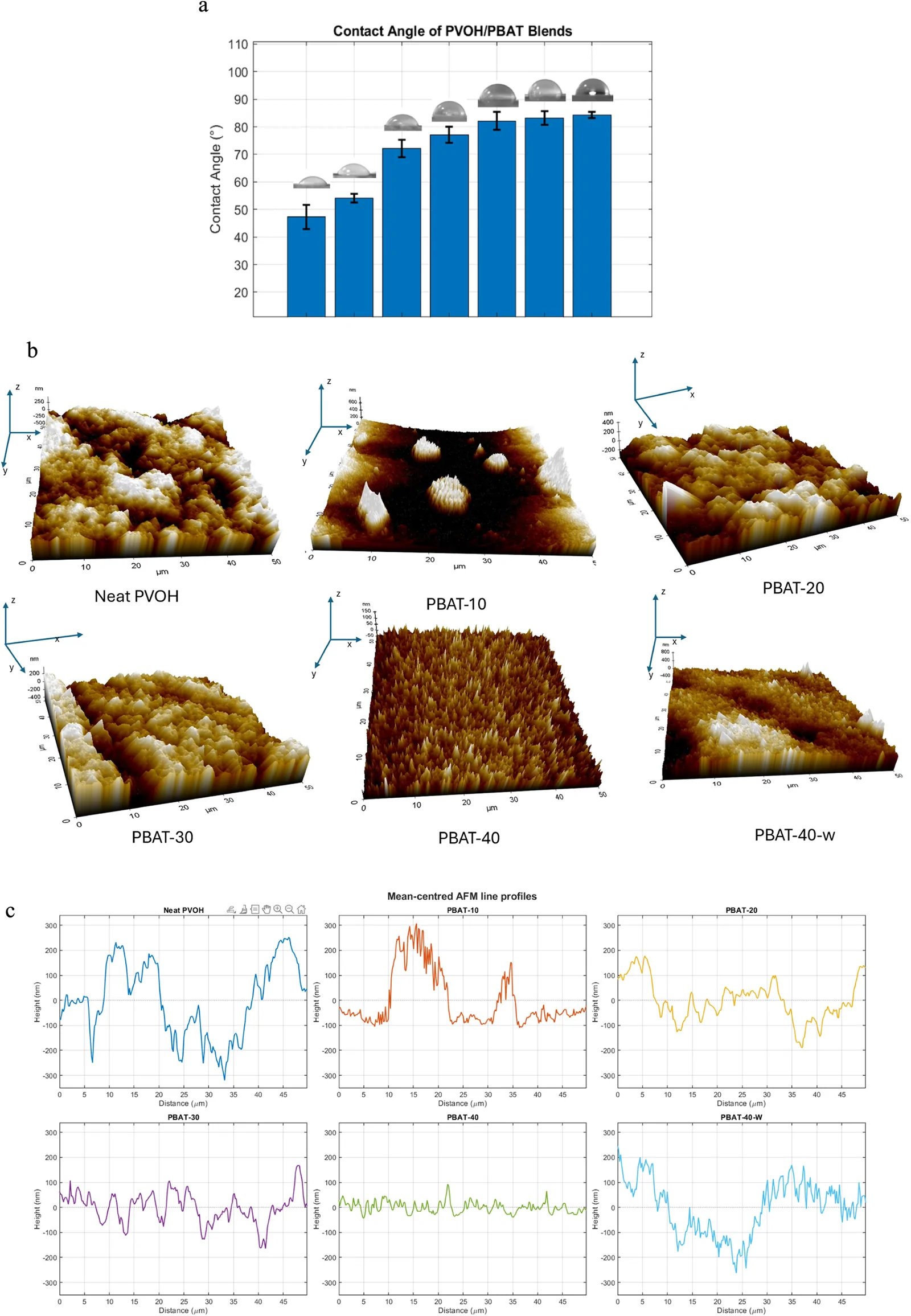
(a) Static water contact angle of the neat polymers and blends (means ± SD; five locations per film, 23 ± 2 °C). (b) AFM three-dimensional height images of the neat PVOH film and the blends (40–50 μm scan size), with the x, y and z axes labeled in μm (x, y) and nm (z). (c) Mean-centered height profiles extracted along a representative line from each scan in (b), plotted on a common vertical scale so that the compositions can be compared directly; the corresponding areal roughness parameters are listed in Table 6.

The morphology evolves with composition. At low PBAT, PBAT-10 is a smooth, plasticized matrix carrying a few isolated, tall protrusions consistent with small dispersed PBAT-rich domains; PBAT-20 shows more uniformly distributed rounded clusters, and PBAT-30 a dense, fine, homogeneous asperity texture. PBAT-40 is both the smoothest and the most uniform surface in the series (Sa = 16.3 ± 2.0 nm, Sz = 117.3 ± 3.7 nm), a homogeneous field of interconnected nanoscale hills and valleys without large aggregates or discrete phase-separated domains; this continuous topography indicates good interfacial compatibility between the PBAT-rich and plasticized PVOH-rich phases and is consistent with the cocontinuous structure seen by SEM (Section 3.8). Its very small roughness scatter (±2.0 nm on Sa) shows that this smooth texture is reproduced across the film rather than confined to isolated regions. The water-only control PBAT-40-W behaves quite differently at the same composition: its mean roughness is 7-fold higher (Sa = 116.3 ± 97.0 nm, Sz = 315.0 ± 129.2 nm) and its scatter is larger than that of any other material, so the surface is not only rougher but heterogeneous from one scan area to the next. The corresponding line profile in Figure 7c is dominated by long-wavelength undulations of ±200 nm, whereas that of the ternary PBAT-40 varies by less than ±50 nm over the same scan length. This is the signature of a coarsely phase-separated surface with weaker interfacial compatibility, consistent with the void-laden, poorly bonded interfaces of this control (Section 3.8).^37,38^

The matched pair separates the two contributions to the wettability of Section 3.9. Across the ternary series the surface smooths as PBAT increases while the contact angle rises, so for these intrinsically hydrophilic surfaces (θ < 90°) chemistry and topography act in the same direction, and Wenzel amplification cannot be excluded from the composition series alone.^39^ PBAT-40 and PBAT-40-W resolve it, because they share a composition and differ only in plasticizer: their areal roughness differs 7-fold (Sa = 16.3 ± 2.0 nm against 116.3 ± 97.0 nm) yet their contact angles are indistinguishable (82.1 ± 3.2° against 83.1 ± 2.4°). A 7-fold change in roughness therefore produces no measurable change in wettability at fixed surface chemistry, which isolates hydroxyl availability as the controlling variable. Consistently, neat PVOH is at once the roughest surface and the most wettable, which reflects its dense surface hydroxyls rather than its topography. Surface chemistry which is set by the declining hydroxyl availability as PBAT replaces PVOH, therefore governs the wettability, with topography a secondary modifier, in agreement with the monotonic contact-angle trend.^40^

### 3.11 Thermal Stability and Degradation Pathways

TGA links the redistributed hydrogen-bond network to the onset of degradation. Neat PBAT is the most stable: negligible loss to ≈330 °C, a single step at 360–430 °C (DTG maximum 405.5 °C), and 4.86 wt % residue at 495 °C. Neat PVOH degrades in stages, with bound-water and low-mass loss above 120 °C (Td5% = 275.3 °C), a main step with a DTG maximum at 315.0 °C, and 6.21 wt % residue at 495 °C. The multistage profile of dehydration, side-group elimination and then main-chain scission is evident for PVOH in Figure 8b.^14^

**Figure 8:**
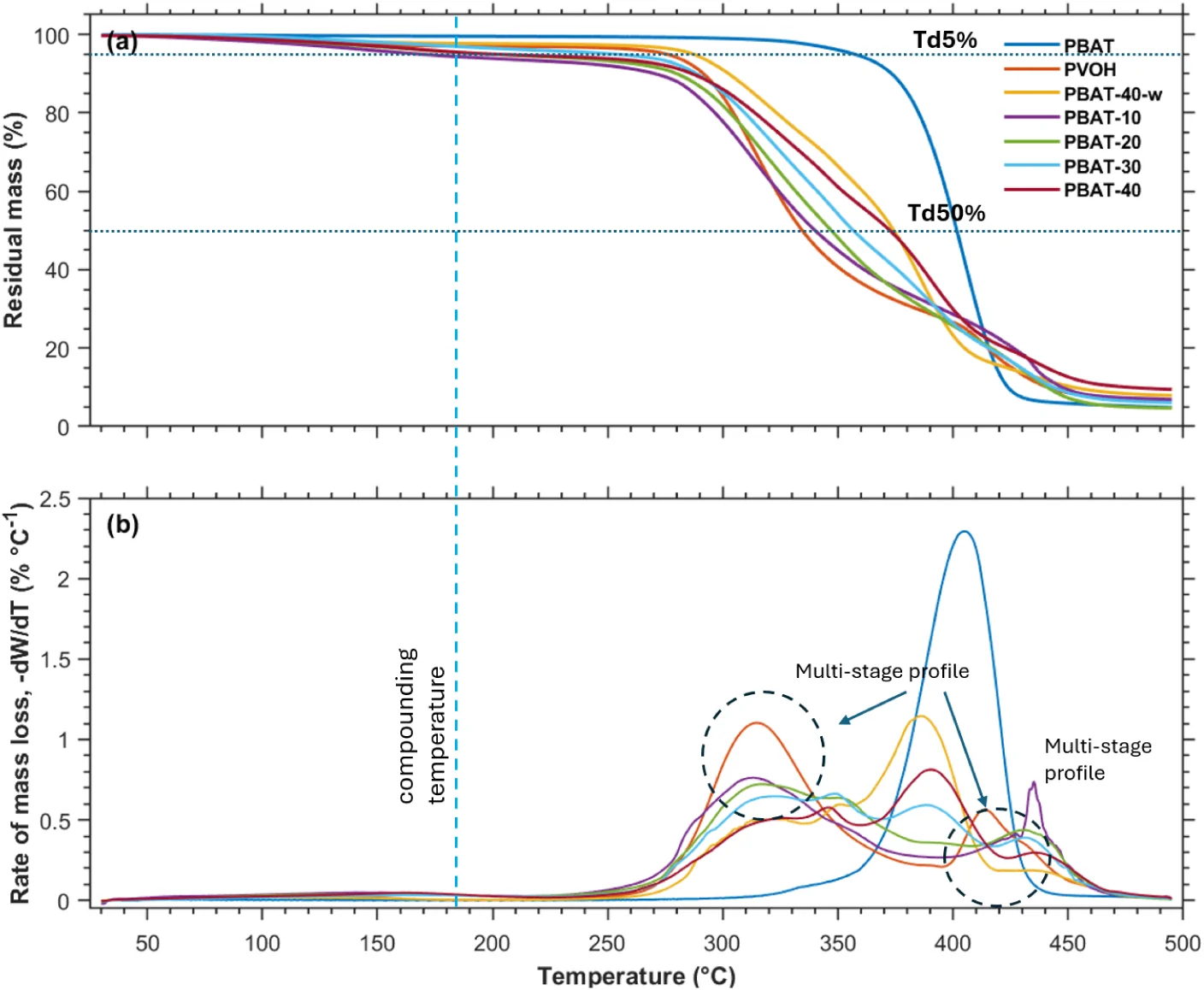
(a) TGA and (b) derivative (DTG) curves (10 °C min^–1^, N_2_) of the neat polymers and blends, plotted on a common temperature axis so that each DTG maximum aligns with the corresponding inflection in the mass-loss curve. Characteristic temperatures are listed in Table 7.

Adding PVOH and plasticizer to PBAT lowers the onset and broadens the envelope. PBAT-20 begins losing mass below 120 °C (moisture and additive volatilization), with a first DTG maximum near 316 °C and a shoulder to 430 °C; PBAT-30 shifts the dominant peak to ≈348 °C with a stronger high-temperature shoulder, the onset remaining far below neat PBAT. The redistributed, amorphous PVOH fraction created by DPE and ε-CL decomposes earlier and overlaps PBAT pyrolysis. PBAT-40-W behaves differently: moisture loss begins near 150 °C, the principal DTG peak is at 386.5 °C, the envelope is narrower, and Td5% is 287.1 °C, some 88 °C above the ternary PBAT-40 at the same composition and the same 20 wt % loading. Because early mass loss reports on the mobile plasticizer fraction rather than on main-chain scission, this separation is the thermal counterpart of the glass-transition result: at 5% loss the two materials differ by 88 °C, whereas at 50% loss they are only 1.7 °C apart (374.2 versus 372.5 °C), by which point the backbone governs both. The higher peak temperature of the water-only control reflects retained PVOH–PVOH association and more compact domains, with dehydration of the intact hydroxyl network promoting char formation as shown in Figure 8a, again separating water-only plasticization from the ternary system, which lowers thermal stability by amorphizing the PVOH phase. Char yield does not follow the same order: the ternary PBAT-40 leaves the most residue (9.47 wt %) against 7.91 wt % for PBAT-40-W, which is consistent with the retained, hydroxyl-rich DPE contributing to char.

Characteristic degradation temperatures are collected in Table 7. Td5% separates the two plasticization routes most clearly, because early mass loss in these materials is dominated by desorption of bound water and volatilization of the mobile plasticizer fraction rather than by main-chain scission.

**Table 7:** Characteristic Thermal-Degradation Parameters from TGA and DTG (10 °C min^–1^, 30–500 °C, N_2_): Temperature at 5% Mass Loss (Td5%), Temperature at 50% Mass Loss (Td50%), Temperature of Maximum Mass-Loss Rate (DTG Max), and Residual Mass at 495 °C, the End of the Measured Range^a^

Sample	Td5% (°C)	Td50% (°C)	DTG max (°C)	Residue at 495 °C (wt %)
Neat PBAT	356.9	401.6	405.5	4.86
Neat PVOH	275.3	334.5	315.0	6.21
PBAT-10	167.7	340.0	313.0	6.88
PBAT-20	197.4	347.0	317.5	4.71
PBAT-30	252.3	356.6	348.5	6.11
PBAT-40	199.2	372.5	390.5	9.47
PBAT-40-W	287.1	374.2	386.5	7.91

a The Td5% value for PBAT-10 is unaffected by the instrument artifact near 435 °C described in the caption to Figure 8; its residue is quoted as measured.

### 3.12 Representative Barrier Performance

The melt-processed PBAT-20 film, with an average thickness of 400 μm, showed moderate barrier performance at 23 °C and 50% RH, with an oxygen transmission rate (OTR) of 6.40 cm^3^ m^–2^ day^–1^ and a water-vapor transmission rate (WVTR) of 6.06 g m^–2^ day^–1^, calculated as the mean values from three films. When thickness is considered, the OTR corresponds to a thickness-normalized value of 6.40 × 400 = 2.56 × 10^3^ cm^3^ μm m^–2^ day^–1^. Alternatively, when normalized to a standard 25 μm film thickness, the equivalent OTR is 6.40 × (400/25) = 102.4 cm^3^ m^–2^ day^–1^, as illustrated in Figure 9. This indicates that PBAT-20 provides moderate oxygen-barrier performance, comparable to some biodegradable PLA/PHBV-type barrier materials,^41,42^ but it does not approach the much lower OTR values typically associated with dense crystalline PVOH or EVOH,^43,44^ which exhibit substantially higher oxygen-barrier efficiency. Two qualifications follow. First, the measured OTR refers to a 400 μm film, an order of magnitude thicker than conventional flexible packaging, so the thickness-normalized value of ≈102 cm^3^ m^–2^ day^–1^ at 25 μm is the appropriate basis for comparison with commercial films. Second, the WVTR of 6.06 g m^–2^ day^–1^ is high for a packaging film. The performance envelope reported here therefore supports oxygen-sensitive but moisture-tolerant contents, such as crisps, dried fruits, nuts, powders, sachets, and secondary or nonfood wrapping, and does not extend to fresh produce, high-moisture foods, or applications requiring extended shelf life under humid conditions.

**Figure 9:**
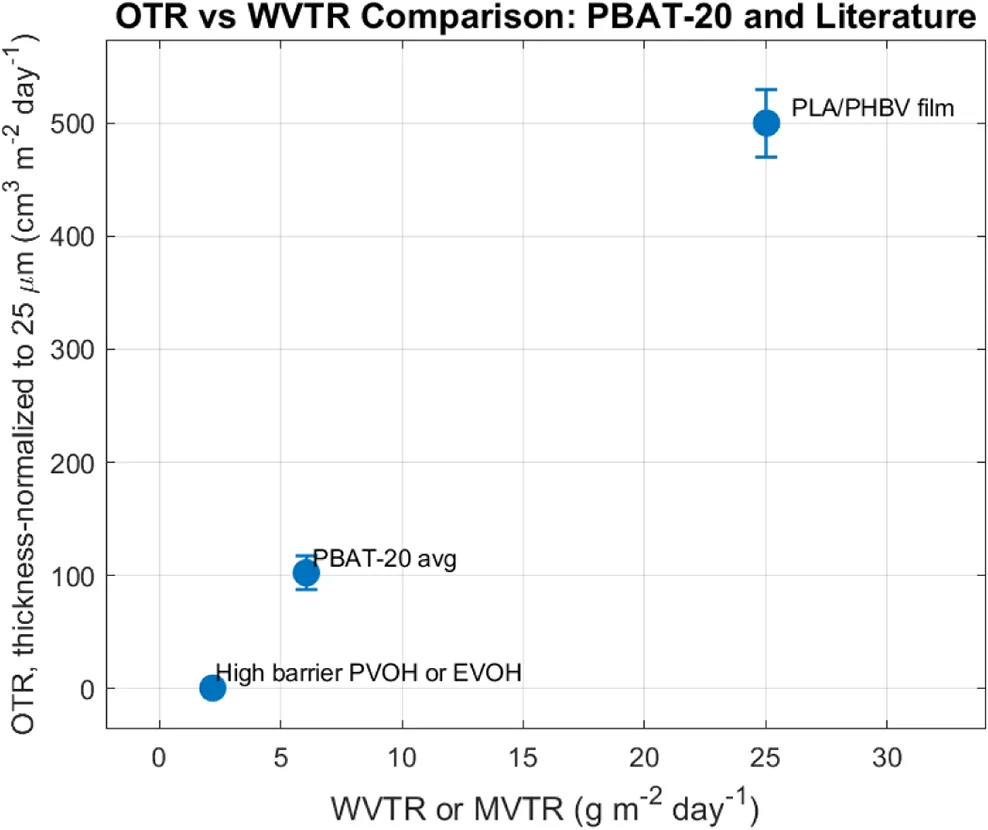
Oxygen transmission rate (OTR) plotted against water-vapor transmission rate (WVTR) for the PBAT-20 film (400 μm; 23 °C, 50% RH; *n* = 3), with the OTR normalized to a 25 μm equivalent thickness so that it can be compared with conventional packaging films. Reference points for a high-barrier PVOH or EVOH film and for a PLA/PHBV film are shown for context; the full comparison with the literature is given in Table 8.

The barrier level is itself a plasticization effect. Adding small hydrogen-bonding molecules like water, glycerol, or polyols, raises chain mobility and free volume in PVOH, which lowers Tg and increases gas diffusion, so higher plasticizer loading trades barrier for flexibility.^6,11^ PBAT-20s moderate barrier is therefore the expected consequence of a free-volume increase that accompanies the same hydrogen-bond redistribution responsible for its processability and ductility. Reinforcement or thinner high-barrier layers (e.g., oriented PVOH or coated architectures) can recover oxygen barrier where the application requires it, at the cost of some of the processing latitude gained by plasticization.^45,46^

Table 8 places these values in the context of comparable PBAT/PVOH and plasticized PVOH systems. Two points emerge from the comparison. First, systems that achieve markedly higher tensile strength do so at high PVOH content or through orientation during film blowing, both of which restrict ductility, whereas the present films retain elongations of one to two hundred percent at a strength appropriate to flexible packaging. Second, barrier data for melt-processed PBAT/PVOH blends are reported infrequently and rarely with the film thickness stated, which limits direct comparison; the values in this work are therefore quoted both as measured and normalized to 25 μm.

**Table 8:** Comparison of the Present PBAT/PVOH Films with Representative PBAT/PVOH and Plasticized PVOH Systems Reported in the Literature^a^

System	Processing	Plasticizer/compatibilizer	UTS (MPa)	εb (%)	OTR (cm^3^ m^–2^ day^–1^) [thickness]	WVTR (g m^–2^ day^–1^)	Refs
**PBAT-20 (this work)**	Melt compounding + compression molding	DPE/ε-CL/water (20 wt %)	**20.2 ± 0.7**	**238.7 ± 28.1**	**6.40 [400 μm]; ≈102 at 25 μm** ^**4**^	**6.06**	–
**PBAT/PVA,20/80wt %**	Twin-screw melt blending + injection molding	None reported	**53.48**	**≈12** ^**1**^	n.a.	n.a.	^1^
**PVA/PBAT blown film,75/25wt %**	Melt extrusion + film blowing	Water/glycerin-plasticized PVA	**44.0**	n.r.	**≈615 [80 μm]**	n.r.	^16^
**Plasticized PVOH film, 20 wt % polyol**	Melt processing	Polyol plasticizer	**≈65–70**	**≈450**	n.r.	n.r.	^12^
**PVOH/DES film, Mowiol 20–98/CA**	Solution casting	ChCl/citric-acid DES; PVA:DES = 70:30 w/w	**51 ± 5**	**362 ± 58**	**≈0.12** ^**1**^	**26.15**	^18^
**PVA/starch/CMC/walnut-shell flour, PSC10W2**	Solution casting	Glycerol 20 wt %	**35.61 ± 0.37**	**≈200**	n.r.	n.r.	^19^
**PVA/casein +15 wt % sorbitol**	Solution casting	Sorbitol, 15 wt %	**≈27** ^**1**^	**369**	n.r.	n.r.^5^	^20^
**PVA/glycerol, PGL (75/25wt %)**	Solution casting	Glycerol, 25 wt %	**≈11** ^**1**^	n.r.	n.r.	**≈624** ^**6**^	^21^

a Barrier values are quoted with the film thickness at which they were measured, since transmission rate scales inversely with thickness; N.D. = not determined in this work, N.A. = not applicable to the specimen geometry.

### 3.13 Structure–Processing–Property Synthesis

The techniques above converge on a single causal sequence, set out here so that the structure–processing–property relationship is illustrated in Figure 10. The chain begins with the hydroxyl population of PVOH. FTIR shows that the ternary plasticizer broadens and shifts the O–H stretching envelope and weakens the crystallinity-sensitive 1141 cm^–1^ band without introducing new covalent bands (Section 3.1), so PVOH–PVOH contacts are exchanged for PVOH–plasticizer contacts rather than destroyed. Fewer PVOH–PVOH contacts remain available for chain packing, and XRD and DSC record the consequence: broadened, weakened PVOH reflections, smaller apparent crystallite sizes, and suppressed recrystallization on second heating (Sections 3.2 and 3.3). The same loss of physical cross-linking raises segmental mobility, which appears thermally as a glass transition depressed by 17–27 °C and mechanically as a depressed, broadened α-relaxation (Sections 3.3 and 3.6). In the melt, that mobility lowers the low-frequency complex viscosity by more than an order of magnitude relative to neat PVOH and broadens the relaxation-time distribution (Sections 3.4 and 3.5), which is what renders the blend melt-processable at 185 °C without entering the decomposition range of PVOH.

**Figure 10:**
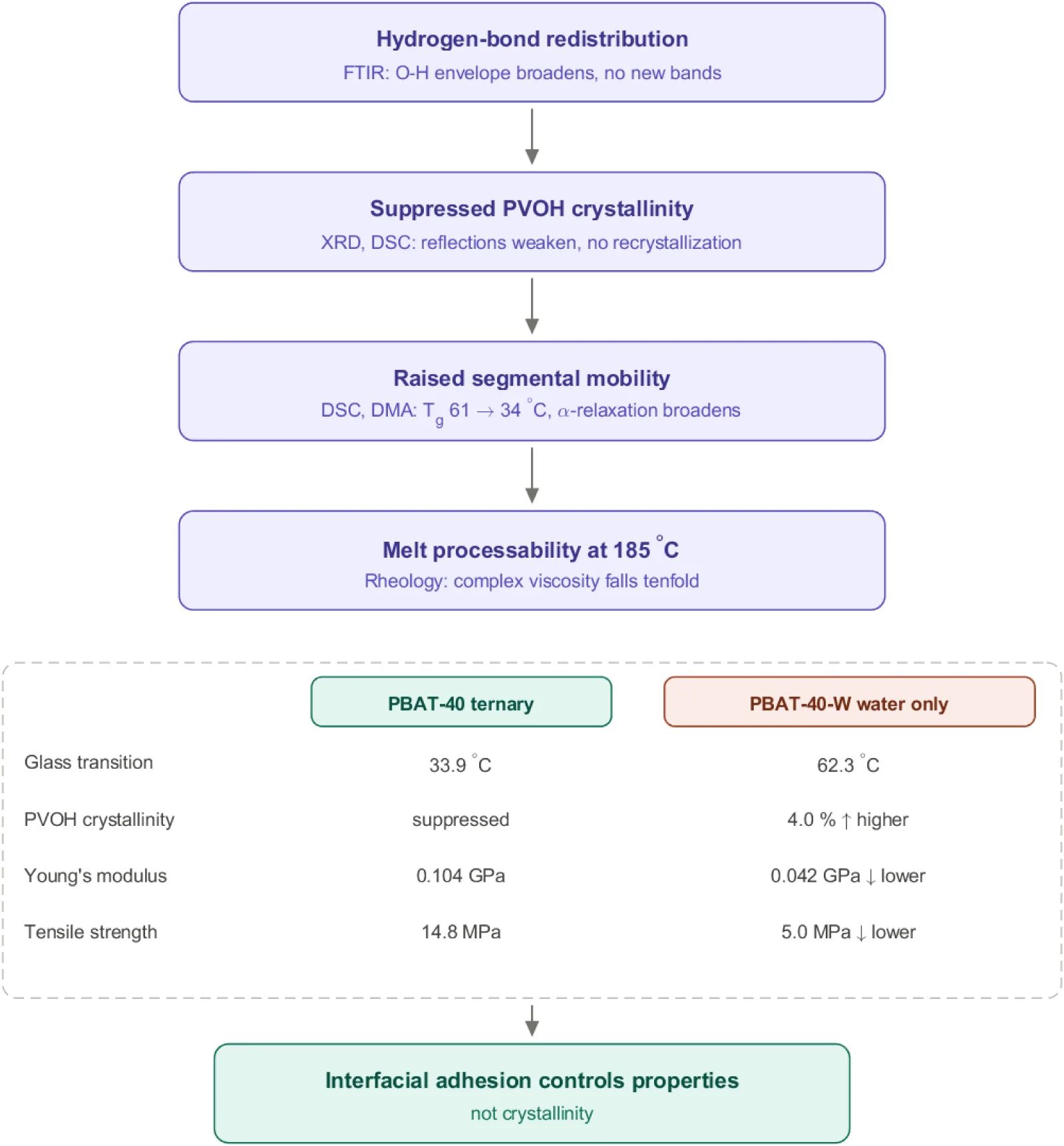
Mechanism linking hydrogen-bond redistribution to melt processability and mechanical performance in the ternary-plasticized PBAT/PVOH blends.

Two variables then act independently on the solid film and separating them is the key to interpreting the mechanical data. Composition sets the phase topology: PBAT forms discrete domains in a continuous PVOH matrix at 20–30 wt % and a cocontinuous structure at 40 wt %, where the polymer ratio reaches 50:50 (Section 3.8). The plasticizer sets the interfacial adhesion at any given topology, as the matched pair PBAT-40 and PBAT-40-W demonstrates at fixed composition and fixed 20 wt % loading. This separation resolves what would otherwise be an ambiguity in the tensile data. Reduced crystallinity alone would depress stiffness and strength together, yet removing DPE and ε-CL lowers the modulus from 0.104 to 0.042 GPa and the strength from 14.8 to 5.0 MPa while the water-only control simultaneously retains the higher crystallinity and the higher glass transition of the pair (Tg, onset 62.3 °C versus 33.9 °C, Table 3). Crystallinity and mechanical performance therefore move in opposite directions across this pair, which isolates interfacial adhesion, rather than crystallinity, as the property-controlling variable.

The water-only control also marks the limit of conventional plasticization. Its glass-transition onset of 62.3 °C is indistinguishable from that of neat PVOH (60.9 °C) within the measured scatter, so water alone produces essentially no solid-state plasticization at the same total loading, and its melt viscosity remains at the level of PBAT-20 rather than falling to that of PBAT-40 (Section 3.5). What water does not do is bridge the two phases: the cryo-fracture surfaces of PBAT-40-W show partial wetting, interfacial debonding, and voids (Section 3.8), and its surface is 7-fold rougher than that of the ternary PBAT-40 at the same composition (Sa = 116.3 against 16.3 nm) and far more heterogeneous between scan areas, in place of the smooth, uniform topography of the ternary blend (Section 3.10). The nonvolatile, six-hydroxyl DPE and the ester-bearing ε-CL supply both the persistent bulk redistribution and the interfacial coupling that water cannot, and the cost of that coupling is visible in the thermal and barrier data: the amorphized PVOH phase decomposes earlier and over a broader envelope than in the water-only control (Section 3.11), and the additional free volume raises gas permeability (Section 3.12).

Hydrogen-bond redistribution by the ternary plasticizer converts PVOH from a solvent-cast polymer into a melt-processable one and simultaneously creates a well-adhered PBAT–PVOH interface, while PBAT content determines how that interface is arranged in space. PBAT-20 is the composition at which the two controls are best matched: the PVOH network remains continuous and load-bearing, the dispersed PBAT domains are dense enough to dissipate energy (Section 3.7), and the oxygen barrier contributed by the PVOH-rich matrix has not yet been diluted by a cocontinuous PBAT network.

## 4 Conclusions

In this work, melt-compounded PBAT/PVOH blends requiring no aqueous casting step were developed, enabled by a ternary plasticizer of dipentaerythritol, ε-caprolactone, and water in equal parts that makes the otherwise poorly processable PVOH melt-blendable by redistributing its hydrogen-bond network rather than by chemical reaction. FTIR, XRD, DSC, DMA, and rheology characterization consistently showed a broadened O–H stretching envelope, suppressed PVOH crystallinity and recrystallization, a glass transition lowered by 17–27 °C, and a broadened relaxation spectrum, with no indication of cross-linking or covalent grafting. PBAT content controls the phase topology, giving discrete PBAT domains in a continuous PVOH matrix at 20–30 wt % and a cocontinuous structure near the 50:50 polymer ratio at 40 wt %, while the ternary plasticizer enhances interfacial adhesion between the PBAT and PVOH phases to give a more uniformly plasticized blend.

For packaging, the 20 wt % PBAT blend (PBAT-20) is the most useful composition among the blends investigated. It combines the best mechanical balance of the series, an ultimate tensile strength near 20 MPa and a Young’s modulus near 0.23 GPa, with an oxygen transmission rate of 6.40 cm^3^ m^–2^ day^–1^ for a 400 μm film at 23 °C and 50% RH, equivalent to ≈102 cm^3^ m^–2^ day^–1^ at 25 μm, which places it within the range reported for biodegradable barrier films. PBAT-20 thus combines the stiffness and oxygen barrier contributed by PVOH with the ductility and melt processability contributed by PBAT in a single melt-processed, biodegradable film. On the evidence presented here the strategy is applicable to oxygen-sensitive, moisture-tolerant packaging formats; extension to moisture-critical food-contact applications would first require the water-vapor barrier to be addressed, as set out below.

The main limitation of this work is the water-vapor barrier. The hydrophilic, PVOH-rich matrix gives a comparatively high water-vapor transmission rate (6.06 g m^–2^ day^–1^), and the free volume introduced by plasticization, the same effect that enables melt processing, works against moisture resistance. Future work will address this by tailoring the ternary plasticizer, shifting its balance toward more hydrophobic, nonvolatile constituents and optimizing the plasticizer loading against barrier, and by adding complementary measures such as compatibilized blending with hydrophobic polymers or hydrophobic surface coatings, with the goal of lowering the water-vapor transmission rate while preserving the oxygen barrier and mechanical performance reported here. On this basis, ternary hydrogen-bond engineering provides a reaction-free, melt-processing route to property-tunable PBAT/PVOH films for environmentally friendly packaging.

## Data Availability

The data supporting the findings of this study are contained within the article.
